# Development of a 24-channel 3T phased-array coil for fMRI in awake monkeys: Mitigating spatiotemporal artifacts in ferumoxytol-weighted functional connectivity estimation

**DOI:** 10.1162/IMAG.a.1354

**Published:** 2026-09-03

**Authors:** Joonas A. Autio, Atsushi Yoshida, Yoshihiko Kawabata, Masahiro Ohno, Kantaro Nishigori, Takayuki Ose, Stephen M. Smith, David C. Van Essen, Matthew F. Glasser, Takuya Hayashi

**Affiliations:** Laboratory for Brain Connectomics Imaging, RIKEN Center for Biosystems Dynamics Research, Kobe, Japan; Department of Neuroscience, Washington University Medical School, St Louis, MO, United States; Systems Neuroscience Laboratory, Hokkaido University Graduate School of Medicine, Sapporo, Hokkaido, Japan; Takashima Seisakusho Co., Ltd, Tokyo, Japan; Sumitomo Pharma Co., Ltd, Osaka, Japan; Centre for Integrative Neuroimaging, FMRIB, Nuffield Department of Clinical Neurosciences, University of Oxford, United Kingdom; Department of Radiology, Washington University Medical School, St Louis, MO, United States; Department of Brain Connectomics, Kyoto University Graduate School of Medicine, Kyoto, Japan; Section of Brain Function Information, National Institute for Physiological Sciences, Okazaki, Japan

**Keywords:** macaque, awake, RF coil, fMRI, ferumoxytol

## Abstract

Functional magnetic resonance imaging (fMRI) of awake macaque monkeys holds promise for advancing our understanding of primate brain organization, including humans. However, estimating functional connectivity in awake animals is challenging due to the limited duration of imaging sessions and the relatively low sensitivity to neural activity. To overcome these challenges, we developed a 24-channel 3T receive radiofrequency (RF) coil optimized for parallel imaging of awake macaques. This enabled the acquisition of cross-plane and in-plane accelerated ferumoxytol-weighted resting-state fMRI. The Human Connectome Project-style data processing pipelines were adapted to address the unique preprocessing demands of cerebral blood volume-weighted (CBVw) imaging, including motion correction, functional-to-structural image co-registration, and training a multi-run independent component analysis-based X-noiseifier (ICA-FIX) classifier for removal of structured artifacts. Our CBVw fMRI approach resulted in an elevated contrast-to-noise ratio compared with blood oxygenation level-dependent (BOLD) imaging in anesthetized macaques. However, structured imaging artifacts still contributed more variance to the functional timeseries than neural activity. By applying the ICA-FIX classifier, we achieved highly reproducible parcellated functional connectivity at the single-subject level, with test–retest matrix correlations and subject identification accuracy comparable with those observed in humans. At the group level, we identified dense functional networks with spatial features homologous to those observed in humans. The developed RF receive coil, image acquisition protocols, and data analysis pipelines are publicly available, providing the broader scientific community with tools to leverage these advances for further research.

## 1 Introduction

The macaque monkey serves as a pivotal animal model in elucidating the intricate neuroanatomical and functional arrangements of the primate brain. Functional magnetic resonance imaging (fMRI) has become a fundamental tool in the investigation of default functional organization within both human and non-human primates (NHPs) (Hutchison et al., 2011; Hutchison & Everling, 2012; Smith et al., 2013; Vincent et al., 2007). Notably, comparison between resting-state and task-induced functional connectivity (FC) patterns in humans has also revealed a high degree of similarity, suggesting an inherent relationship between task-induced neural networks and resting-state brain activity (Glasser et al., 2018; Nickerson, 2018; Smith et al., 2009). Therefore, resting-state fMRI has the potential to bypass the major challenge of task-design across species, enabling evolutionary comparison of default functional organization among primates (Mantini et al., 2011; Xu et al., 2020; Yokoyama et al., 2021).

Despite the promise of interspecies comparisons, challenges persist due to the relatively limited sensitivity and specificity of fMRI, compounded by factors such as hardware constraints, anesthesia effects, and a lack of standardized data preprocessing pipelines, leading to reduced reproducibility in NHP resting-state fMRI studies (Autio et al., 2021; Botvinik-Nezer et al., 2020; Milham et al., 2018). To bridge this gap, recent advances have adapted human neuroimaging methodologies to NHPs, including the development of a specialized 3T multi-channel radiofrequency (RF) receive coils, facilitating the translation of imaging and data analysis strategies from the Human Connectome Project (HCP) to anesthetized NHPs (Autio et al., 2020; Donahue et al., 2018; Glasser et al., 2013; Hayashi et al., 2021; Ose et al., 2022; Smith et al., 2013). This neuroanatomically informed approach has a potential to significantly enhance our ability to map the structural and functional organization in non-human primates (NHPs) (Yokoyama et al., 2021). However, the influence of anesthesia on functional inter-species comparisons remains a concern (Paasonen et al., 2018; Vanduffel et al., 2001), prompting efforts to extend these NHP–HCP methodologies to awake macaque monkeys.

Imaging awake macaques introduces distinct challenges compared with studies with anesthetized subjects (Vanduffel et al., 2001). Notably, the necessity of surgically mounting a headpost for RF coil attachment alongside an animal chair must be balanced with practical constraints related to scan duration and potential discomfort. These constraints highlight the need for fast, cross-plane accelerated imaging achievable through multiband (MB) imaging (Moeller et al., 2010), to acquire a large number of timepoints necessary for resting-state fMRI. Additionally, scan duration can also be eased by the application of an ultrasmall superparamagnetic iron oxide (USPIO) contrast agent, ferumoxytol, to boost the contrast-to-noise ratio (CNR) (Leite et al., 2002; Mandeville et al., 1999, 2004; Vanduffel et al., 2001), with optimal brain contrast achieved at the shortest feasible echo time (TE) (Mandeville et al., 1999, 2004). The use of in-plane acceleration, such as GeneRalized Auto-calibrating Partially Parallel Acquisitions (GRAPPA) (Griswold et al., 2002), further facilitates the reduction of TE and repetition time (TR). The combination of in- and out-of-plane accelerations sets stringent demands on the parallel imaging performance of the RF coil (Gilbert et al., 2016).

The subsequent data analysis challenges of accelerated ferumoxytol-weighted fMRI also remain poorly understood. For instance, structured spatiotemporal artifacts impose a challenge that can significantly impact FC estimates (Power et al., 2012; Van Dijk et al., 2012). While the efficacy of independent component analysis-based X-noiseifier (ICA-FIX) artifact removal is well established in human BOLD fMRI studies (Glasser et al., 2018; Griffanti et al., 2014; Salimi-Khorshidi et al., 2014), its efficacy in CBVw fMRI experiments remains unexplored. For instance, ferumoxytol may partially wash out during a functional imaging session, thereby introducing spurious global correlations across the brain. Moreover, it is well established that injection of contrast agents causes strong static magnetic field (B_0_) orientation bias (Autio et al., 2025; Ogawa et al., 1993). Yet, the impact of ferumoxytol to FC metrics remains largely unexplored.

In this study, we introduce a novel 24-channel 3T receive RF coil tailored for parallel imaging of awake macaque monkeys. Capitalizing on the parallel imaging capabilities of the phased-array RF receive coil, we acquire accelerated ferumoxytol-weighted resting-state fMRI in awake macaque monkeys. We explore the effect of ICA-FIX artifact cleanup to CBVw functional connectivity estimation and reproducibility of the within and between macaques. We apply this approach to distinguish individual macaques by their unique neural fingerprints.

## 2 Methods

Experiments were performed using a 3T MRI scanner (MAGNETOM Prisma, Siemens, Erlangen, Germany) equipped with 80 mT/m gradients (XR 80/200 gradient system with slew rate 200 T/m/s) and a 2-channel B_1_ transmit array (TimTX TrueForm). The animal experiments were conducted in accordance with the institutional guidelines for animal experiments, and animals were maintained and handled in accordance with the institutional guidelines for animal experiments, Basic Policies for the Conduct of Animals Experiments in Research Institution (MEXT, Japan), and the Guide for the Care and Use of Laboratory Animals of the Institute of Laboratory Animal Resources (ILAR; Washington, DC, USA). All animal procedures were approved by the Institutional Animal Care and Use Committee (IACUC) of RIKEN Kobe Campus (MA2008-03-19, A2021-05-6).

### 2.1 Development of a phased-array 3T receive coil for awake, headpost-implanted macaque monkeys

The 24-channel coil for awake macaque imaging presented here builds upon our previously published design (Autio et al., 2020). However, as noted in the introduction, imaging awake monkeys requires an implanted headpost, a secure attachment mechanism, and an MRI-compatible MRI chair, features that were explicitly incompatible with our previous coil design.

Addressing this limitation was a non-trivial challenge from a coil design perspective. The headpost attachment necessitates a larger opening in the phased-array coil, which can potentially compromise SNR, channel decoupling, and parallel imaging performance. To mitigate these issues, we developed a modular, attachable coil design that accommodates the headpost, while modified coil element reconfiguration maintains high SNR and parallel imaging acceleration capabilities. The attachable configuration enables both stability and flexibility in experimental setups while largely preserving the benefits of a dense phased-array coil for accelerated imaging.

#### 2.1.1 Coil design, construction, and evaluation

**Fig. 1.**
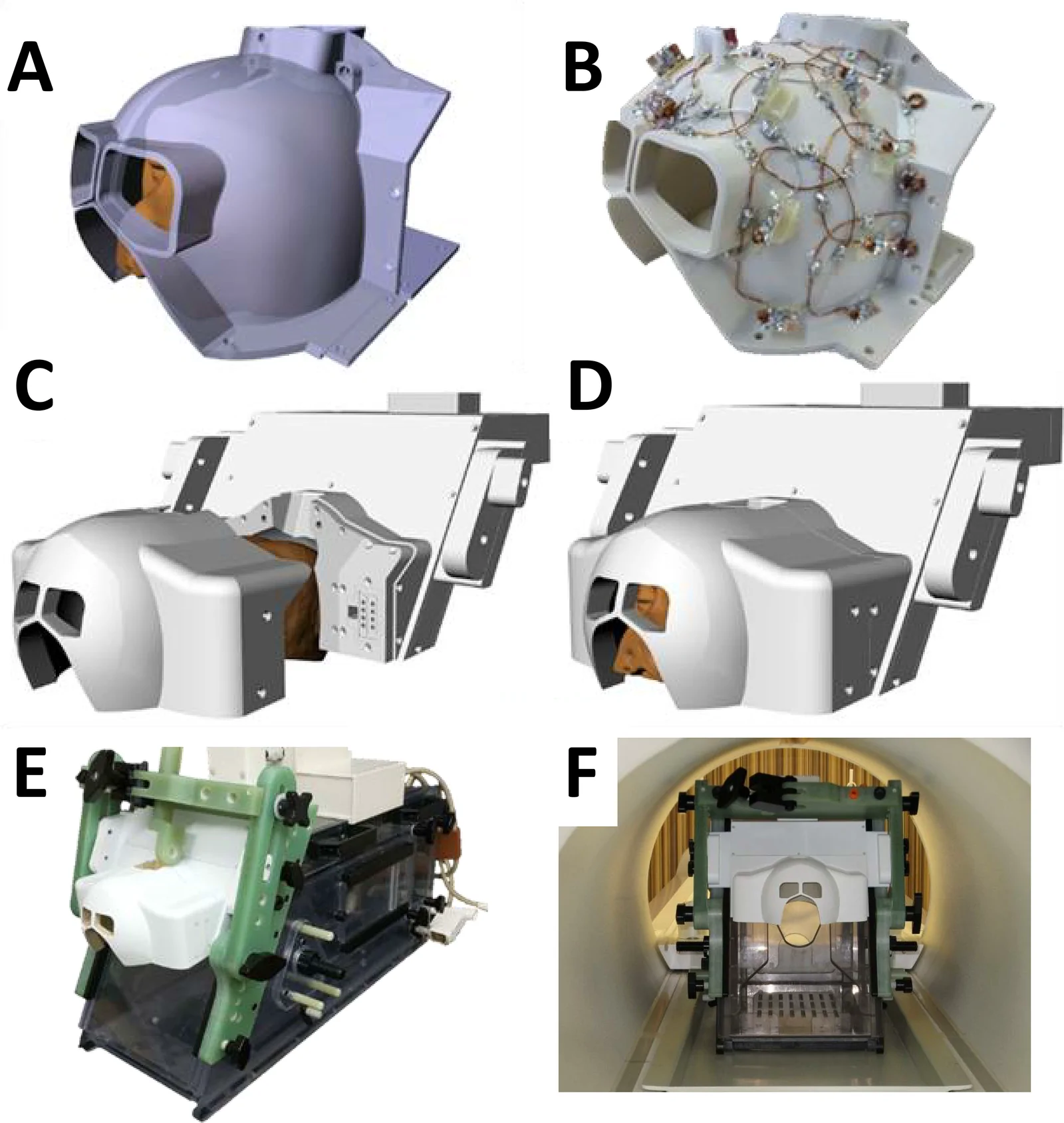
The design and development of a 24-channel receive coil for awake headpost-implanted macaque MRI. (A) Design of coil geometry and (B) coil with 24-element arrangements. (C, D) The coil elements were separated into anterior and posterior halves to easily equip the coil on the monkey. (E) Coil attached to a customized MRI-compatible primate chair (Rogue Research, Mtl, CA, Canada). (F) The primate chair and coil positioned on a customized plastic plate within the bore of the scanner. Note that the chair is placed on the plastic plate rather than on the scanner’s built-in bed.

The coil frame geometry was designed using a 3D digital design software (Rhinoceros5, McNeel, Seattle, USA) to closely fit over the headpost-implanted (*Macaca mulatta*) monkeys (Fig. 1A). The helmet-shaped inner coil frame was constructed from machine-carved polyphenylene sulfide (PPS) plates. The thin PPS plates (2 mm) minimized the distance between coil elements while providing good workability and resistance to the heat of soldering.

Twenty-four coil elements were arranged over external surface, following a soccer ball-type coil design (Wiggins et al., 2006). To reduce potential interaction with RF transmit field (B_1_ +), the elements were wired using thin coaxial copper cables (cable diameter 0.7 mm) and capacitors were arranged vertically against the coil surface (Autio et al., 2020; Wiggins et al., 2009). The coil elements covered the entire monkey’s head, excluding the mouth region, and were arranged with critical overlap to minimize coupling between neighboring elements (Supplementary Fig. S1). This design improved decoupling between coil elements, which is essential for optimizing parallel encoding performance of accelerated MRI sequences.

For ease of placement, the coil was designed into two modular halves (anterior and posterior) (Fig. 1C, D). A novel feature of the design is that the elements at the interface between the two halves were overlapped by positioning the inductors contained within each element facing each other across the front and posterior halves, allowing the users to easily and firmly fix the head and two halves of coils while achieving efficient decoupling between corresponding elements (Fig. 1B, C). In addition, two elements positioned over the eyes were made slightly larger in diameter to permit video unobstructed recording of eyes and eyelids for behavioral state assessment (e.g., alertness vs drowsiness).

The outer cover (e.g., the frame of the coil; Fig. 1D) was designed for easy attachment to an MRI-compatible monkey chair (HypexInnovations, Rogue research, Inc., Canada) (Fig. 1E) and constructed using acrylic-based resin and a 3D printer (M200, Zortrax, Olsztyn, Poland). The chair was positioned on a custom-made plastic plate/holder, rather than being placed directly on the MRI bed (Fig. 1F). Overall, the resulting central location of the macaque brain (e.g., thalamus) was positioned approximately 10 cm above the B_0_ isocenter.

Circuit design followed standard design consisting of diode detuning trap, cable trap (to achieve common suppression for each element), and bias T connected to low-input impedance preamplifiers (Siemens Healthcare, Erlangen, Germany) (Wiggins et al., 2006; Autio et al., 2020). Elements were tuned to the 3T Larmor frequency (123.2 MHz) by adjusting the trimmer capacitors. Impedance matching was accomplished through fine adjustment of inductor and capacitor values to achieve a reflection coefficient better than -20 dB. Tuning and matching were performed by S11 measurements using a saline phantom placed inside the coil.

The coil preamplifier (MPM series, Microwave Technology Inc.,CA, USA) was positioned above the monkey chair (Fig. 1E), with a cable length of 0.85 m from the coil to the preamplifier. Ideally, the preamplifier would be positioned adjacent to the coil to maximize SNR. However, such a configuration would significantly increase the coil size, making it impractical for experimental use. Pilot studies demonstrated that increasing the distance between the coil and preamplifier from directly adjacent to 1.5 m results in a 20% SNR reduction. To balance signal preservation with usability, we optimized the cable length to 0.85 m, corresponding to half of the RF wavelength specific to the cable material. This adjustment ensured impedance matching and phase consistency between the coil and preamplifier, minimizing signal loss.

Decoupling was achieved through a combination of geometrical and preamplifier decoupling. The latter was implemented by maintaining a very low impedance level in both the coil and at the preamplifier inputs. To ensure consistent impedance and phase alignment, the cable length was set to half the cable-specific RF wavelength (λ), optimizing decoupling efficiency and preserving the performance of the 24-channel receive coil. Geometrical decoupling was assessed using S21 measurements, and overlaps were iteratively adjusted during assembly to meet decoupling target (≤ -20 dB). Active detuning was implemented using a PIN-diode-based detuning trap consisting of a diode, capacitor, and inductor. Detuning efficiency was evaluated using S21 measurements with a 5-mm pickup probe to ensure detuning efficiency better than -20 dB. Tuning, matching, geometrical decoupling, and active detuning steps were iterated to ensure performance across all elements. Although preamplifier decoupling isolation values were not measured separately, the array was configured with low-input impedance preamplifiers connected via λ/2 cables.

The coil elements were assessed for the ratio of loaded to unloaded quality factor Q, nearest-neighbor coupling, and active detuning, as well as gradient off-line noise correlation. The Q-ratio was assessed using a Vector Network Analyzer (E5061B, KeySight Technologies, CA, US) and a tightly fitted phantom (solution NiCl + NaCl) used for loading. The B_1_ transmit (B_1_ +) field map was estimated using a slice-selective RF-prepared sequence with a turbo fast low-angle-shot (FLASH) read out (Chung et al., 2010). The B_1_ receive (B_1_-) field map was estimated using a FLASH sequence, based on the signal intensity ratio between phased-array and body receive coils.

In-plane geometry-dependent noise amplification due to parallel imaging (g-factor) was assessed using gradient-echo imaging and GRAPPA and a phantom (Griswold et al., 2002). A Fast Low Angle SHot (FLASH) GRE sequence was used with the following parameters: voxel size 1.3 × 1.3 × 2.0 mm, FOV 120 × 120, matrix size 96, coronal orientation, PE-direction FH, full k-space, slices 35, iPAT = 0, 2, 3, 4, 5, 6, 7,8, number of reference lines 32, TE 3 ms, TR 200 ms, and number of scans 50. To correct for image distortion, a GRE B_0_ field map was acquired using voxel size 1.3 × 1.3 × 1.3 mm, FOV 130 × 130, matrix size 100, coronal orientation, PE-direction FH, full k-space, slices 63, bandwidth 417 Hz/Px, TE1 5.1 ms, TE2 7.5 ms.

The g-factor associated with MB, and combined MB+GRAPPA (Preibisch et al., 2015), was evaluated using phantom and GRE EPI sequence with the following parameters: voxel size 1.25 × 1.25 × 1.25 mm, FOV 110 × 110 mm, matrix size 88 × 88, coronal orientation, PE-direction RL, partial Fourier 7/8, slices 60, iPAT 0, 2, 3, MB factors 0, 2, 3, multiband LeakBlock kernel on (Cauley et al., 2014; Risk et al., 2018), TE 31.6 ms, TR 4700-1180 ms, FA 88-66, echo spacing 0.73-0.76 ms, bandwidth 1624 Hz/px, and number of scans 100. For each MB and GRAPPA combinations, Ernst flip angle was determined using phantom T1-value and (minimum) TR while TE was kept constant. For distortion correction, SE EPI B_0_ field maps were acquired with the same geometric parameters (1.25 × 1.25 × 1.25 mm, FOV 110 × 110, matrix size 88 × 88, 60 slices), using opposing PE directions (RL and LR), partial Fourier 7/8, echo spacing 0.73 ms, and bandwidth 1624 Hz/Px.

#### 2.1.2 Headpost surgery

Macaque monkeys (Macaca mulatta, N=3, weight=5.1 ± 0.8 kg, age=6.3 ± 1.0 y.o.) were trained to sit in a sphinx position in an MRI-compatible primate chair (Rogue Research, Mtl, CA, Canada). Upon successful completion of the training, the animals were anesthetized and T1w images were acquired (see Section 2.2). The images were used to generate a bone contour of the subject’s skull surface (Rogue Research, Mtl, CA, Canada). Then, a custom-made headpost was produced to closely match the contour of each subject’s skull surface using PEEK (Poly Ether Ether Ketone, Rogue Research, Inc. Canada).

The headpost was surgically implanted in a sterile environment. The animals were initially anesthetized with ketamine (7.5 mg/ml/kg, i.m.) and xylazine (1.5 mg/ml/kg, i.m.), intubated and deep anesthesia was maintained using isoflurane (≥1.5%, Apollo anesthesia workstation, Dragerwerk AG & Co., Germany). Prior to surgery, the animals were administered antibiotics (Victus, DS Pharma Animal Health Co., Ltd, Japan; 5 mg/ml/kg, i.m.) and an analgesic (Sosegon, MaruishiPharmaceutical Co., Ltd, Japan; 0.2 ml, i.m.). The subjects’ heads were shaved and disinfected with isodine (10%, Meiji Seika Pharma Co., Ltd, Japan). During the surgery, body temperature was controlled with a heating pad (KN-474-S, Natsume Seisakusho, Co. Ltd, Tokyo, Japan) while physiological parameters (respiration rate, heart rate, oxygen saturation, body temperature, and expired CO_2_) were continuously monitored (FUKUDA COLIN Co., Ltd, Tokyo Japan).

The animal was positioned in a stereotaxic holder (Narishige Group, Tokyo Japan), and the headpost was surgically mounted using electrical scalpel (electrosurgical unit, vendor: Semco Co., Ltd Japan, OR Meiji Seika Pharma Co., Ltd, Japan), ceramic screws (Rogue Research, Mtl, CA, Canada), and dental bond (Super-Bond C&B, Sun Medical Co.,Ltd, Japan), which were then covered with acrylic resin (UNIFAST 2, GC Co., Tokyo, Japan). Following the surgery, the animals received antibiotics (Victus, DS Pharma Animal Health Co., Ltd, Japan; 5 mg/ml/kg, i.m.) and analgesic (Metilon, Daiichio-Sankyo Co.,Ltd, Japan, 0.4-0.5 ml) for 4 days.

### 2.2 Pilot studies

#### 2.2.1 Structural image acquisition

For anatomical reference and headpost surgeries, we acquired structural images using a 24-channel coil designed for anesthetized macaque monkeys (Autio et al., 2020). The macaques were sedated with a combination of atropine (0.2 ml, i.m.), dexmedetomidine (3 µg/kg, i.m.), and ketamine (6 mg/kg, i.m.), after which they were intubated with tracheal tubes. Anesthesia was maintained with intravenous dexmedetomidine (3 µg/kg/hr) and 0.6% isoflurane administered via a calibrated vaporizer with a mixture of air 0.75 L/min and O_2_ 0.1 L/min. Subjects were ventilated (Cato, Drager, Germany), and catheters were inserted into the caudal artery for blood–gas sampling. Rectal temperature was maintained at approximately 38 °C using a blanket.

Structural T1w images were acquired using a 3D MPRAGE sequence with the following parameters: 0.5 mm isotropic, FOV 128 × 128 × 112 mm, matrix 256 × 256 slices per slab 224, coronal orientation, readout direction of inferior (I) to superior (S), phase oversampling 15%, averages 3, TR 2200 ms, TE 2.2 ms, TI 900 ms, FA 8.3, bandwidth 270 Hz/pixel, GRAPPA 2, turbo factor 224, and pre-scan normalization. T2w images were acquired using a 3D SPACE sequence with the following parameters: 0.5 mm isotropic, FOV 128 × 128 × 112 mm, matrix 256 × 256, slice per slab 224, coronal orientation, readout direction I to S, phase oversampling 15%, TR 3200 ms, TE 562 ms, bandwidth 723 Hz/pixel, GRAPPA 2, turbo factor 314, echo train length 1201 ms, and pre-scan normalization.

After headpost implementation, all structural image acquisitions were performed using the newly developed 24-channel coil optimized for awake imaging. The imaging parameters remained the same as above, except for the orientation was transversal and phase encoding direction RL.

#### 2.2.2 Quantitative relaxation mapping

To estimate ferumoxytol content in the brain vascularity, quantitative transverse relaxation time (T_2_*) mapping was performed before and after each ferumoxytol injection, as well as at the end of each session to assess potential washout of the ferumoxytol contrast agent. T_2_*-maps were generated using a 3D multi-echo gradient-echo sequence with 1.25 mm isotropic resolution and TEs of 2, 6, 10, 14, 18, and 22 ms. Other imaging parameters were FOV 80 × 80 × 80 mm, matrix 64 × 64 × 64, phase partial Fourier 7/8, bandwidth 610 Hz/pixel, flip-angle 10°, GRAPPA 2, and TR 50 ms. The scanners image reconstruction and multi-echo fitting algorithm were used to generate quantitative T_2_* (=1/R_2_*) images. In rare cases where the contrast agent injection failed, the experiment was either cancelled (for awake macaques), or an additional dose of ferumoxytol was administered (for anesthetized animals) to achieve target T_2_*-value in gray matter.

To determine the Ernst angle for resting-state fMRI, semiquantitative longitudinal relaxation time (T_1_)-maps were obtained using the MP2RAGE sequence (N=2). The imaging parameters were 0.5 mm isotropic, TR 5000 ms, TE 2.2 ms, TI1 600 ms, TI2 2200 ms, FA1 4°, FA2 4°, bandwidth 270 Hz/pixel, GRAPPA 2 with 24 PE reference lines, turbo factor 168, and pre-scan normalization.

#### 2.2.3 Evaluating accelerated imaging

We evaluated the efficiency of multiband slice acceleration factor on fMRI tSNR in anesthetized macaque monkeys. In brief, simultaneous slice excitation with increasing multiband factors reduces the TR, resulting in incomplete T_1_ recovery and a consequent decrease in the optimal (Ernst) flip-angle and tSNR. However, since more data volumes can be acquired within a fixed time window, a more informative measure of data quality is the tSNR scaled by the square root of the number of acquired timepoints (Smith et al., 2013). To account for this, tSNR was evaluated over a matched acquisition time (10 min) across various multiband factors (1, 2, 3, 4, 5, 6, 7, and 8), with corresponding minimum excitation and refocusing RF-pulse lengths (maintaining constant spectral width), minimum TRs, blood Ernst angles, and a fixed bandwidth and TE (=30 ms).

#### 2.2.4 Optimizing ferumoxytol contrast agent dose

After practical in-plane GRAPPA and cross-plane multiband accelerations were determined, we next set to determine optimal amount of ferumoxytol contrast agent in brain vasculature (see Appendix). For this objective, we used ferumoxytol contrast agent concentration (0, 6, 12, 18, and 24 mg/kg) on resting-state fMRI fluctuations in anesthetized macaques (N = 3). Between each injection, quantitative T_2_-maps were acquired.

### 2.3 Resting-state fMRI in awake monkeys

For CBVw fMRI, gradient safety limitations (Gradient Safety Watch Dog; Siemens) were disabled to allow stronger slew rates and faster *k*-space coverage, enabling a shorter TE . Additionally, the use of GRAPPA acceleration (factor 2) further contributed to shortening TE (= 15 ms), benefiting from the improved parallel imaging performance enabled by the newly developed coil. The imaging parameters were 1.25 mm isotropic, FOV 90 × 90 mm, matrix 72 × 72, number of slices 48, phase partial Fourier 7/8, bandwidth 1654 Hz/pixel, echo spacing 0.73 ms, TR 755 ms, flip angle 55°, multiband factor 3, multiband LeakBlock kernel, GRAPPA 2 with a 30 s gradient-echo reference scan to reduce physiological noise in the image reconstruction (Polimeni et al., 2016; Vu et al., 2017), and pre-scan normalization. To determine optimal contrast agent dose, CBVw resting-state fMRI pilot experiments were performed in anesthetized macaque monkeys using varying doses (0, 6, 12, 18 and 24 mg/kg) of ferumoxytol (Feraheme, AMAG Pharmaceuticals Inc, MA, USA).

The B_0_ field map was estimated using a pair of spin-echo EPI images with opposite phase encoding directions (Andersson et al., 2003) (LR and RL directions, 1.25 mm isotropic, TE 46 ms, TR 4.4 s, prescan normalization). The spin-echo EPI scans were matched with geometry and distortion properties of fMRI acquisitions (FOV 90 × 90 mm, matrix 72 × 72, number of slices 48, phase partial Fourier 7/8, bandwidth 1654 Hz/pixel, echo spacing 0.73 ms, GRAPPA 2, fat-suppression, flip angle 90°, averages 4).

For functional imaging of awake macaques, the subject’s head was securely fixed to the coil using a headpost, and the monkeys were trained to sit in a head-first-sphinx position in an MRI-compatible monkey chair (Fig. 1E, F). The monkeys were gradually accustomed to the auditory noise of the MR pulse sequences (e.g. echo-planar imaging) and the mock-scanner environment (by MO). Once the training was successfully completed, functional image acquisition was performed using a protocol similar to the CBVw fMRI protocol described above, with the slice orientation set to the coronal direction in scanner coordinates (corresponding to the axial direction in brain coordinates). Each scan session consisted of 4 single-run scans, each lasting 10 min, resulting in a total of 3,116 frames and an overall acquisition time of 40 min. Monkeys exhibited no signs of discomfort during the imaging sessions. On the contrary, monkeys had a tendency to become drowsy during imaging sessions. To help maintain arousal levels, subjects were rewarded with apple puree between the 10-min fMRI runs. Additionally, an Inscapes video (www.headspacestudios.org), developed with developmental specialists and widely used in pediatric and adult studies, was displayed via an MRI-compatible monitor (BOLDscreen, Cambridge Research Systems Ltd, Kent, UK) to enhance compliance and reduce head movement and drowsiness (Vanderwal et al., 2015, 2019). The Inscape evokes FC patterns more similar to rest than conventional movies (Vanderwal et al., 2015).

To further monitor alertness, we continuously tracked the animal’s eyes using an MRI-compatible camera. Although our study was not designed to rigorously quantify alertness levels, we estimate that animals appeared drowsy in approximately 5% of scans, in which case we used the scanner’s loudspeaker to alert them. In fewer than 10% of scans, animals had their eyes closed, though this alone does not necessarily indicate drowsiness. No imaging sessions were excluded due to sleep or excessive motion.

Each imaging session was separated by at least a week to allow washout of ferumoxytol contrast agent. The imaging protocols are publicly available (https://brainminds-beyond.riken.jp/hcp-nhp-protocol).

### 2.4 Data analysis

#### 2.4.1 Coil acceleration performance analysis

Each accelerated image was registered to structural T1w and T2w images using HCP–NHP pipeline (GenericfMRIVolumeProcessingPipelineNHP.sh) and distortion corrected using B_0_ field maps and FSL’s topup using an optimized configuration for macaque fMRI resolution and readout (b02b0_macaque_fMRI.cnf). Scanner gradient heating-related signal drift was removed from repeated FLASH and EPI GRE data using FSL’s high-pass temporal filtering (σ = 200 s). Temporal SNR (tSNR) maps were computed voxel wise from the repeated measurements by dividing the mean timeseries signal intensity by its standard deviation. G-factor maps were derived using the following formula:

$${\text{g=tSNR}}_{\text{full}}{\;/\;(\text{tSNR}}_{\text{acc}}*\;\text{sqrt (R)}),$$ (1)

where tSNR_full_ and tSNR_acc_ are the temporal signal-to-noise ratios of the fully sampled and accelerated acquisitions, respectively, and R is the GRAPPA acceleration factor (Griswold et al., 2002; Breuer et al., 2009). Apparent g-factor was calculated also for MB accelerated data (Preibisch et al., 2015):

$${\text{g}}_{\text{app}}{\text{=tSNR}}_{\text{full}}{\text{ / tSNR}}_{\text{MB}}\text{.}$$ (2)

Since MB and GRAPPA accelerated imaging enables faster TR, a more informative measure tSNR efficiency was computed by dividing the tSNR with square root of TR. Additionally, assuming that the phantom signals are static over scanning period, denoised tSNR efficiency was computed by regressing out structured temporally dynamic components of FSL’s MELODIC ICA followed by minimal spatial smoothing (σ = 1.25 mm).

#### 2.4.2 Adapting NHP–HCP data analysis pipelines for CBV-weighted resting-state fMRI

**Fig. 2.**
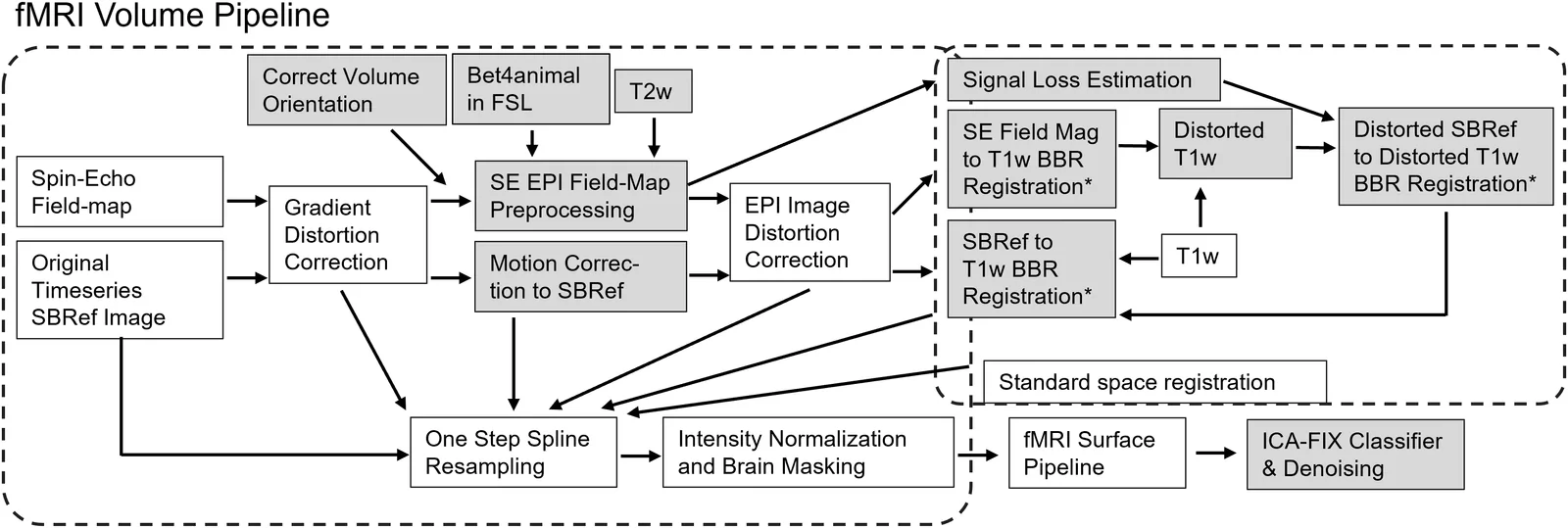
Functional MRI data analysis flow chart. Gray boxes indicate modifications to the previous fMRI pipeline (Autio et al., 2020; Glasser et al., 2013). *BOLD fMRI uses white/gray boundary while CBVw or ferumoxytol-weighted fMRI uses brain outer surface boundary for fMRI to T1w BBR registration. Abbreviations: BBR: boundary-based registration; EPI: echo-planar imaging; ICA: independent component analysis; LR/RL: left*–*right phase encoding directions; MSM: Multimodal Surface Matching; SBRRef: single-band reference scan; and SE: spin echo.

Data analysis was performed using a non-human primate (NHP) adaptation of the HCP pipelines (NHP*–*HCP) (Autio et al., 2020; Glasser et al., 2013; Hayashi et al., 2021), which includes both structural and functional preprocessing. The NHP*–*HCP pipelines utilize FMRB’s Software Library (FSL) version 6.0 (Jenkinson et al., 2012; Smith et al., 2004), FreeSurfer version 6 (Fischl, 2012), and Connectome Workbench version 2.0. These pipelines are available at the HCP pipeline (https://github.com/Washington-University/HCPpipelines), as well as *fmriqc.sh* quality control tools at (https://github.com/RIKEN-BCIL/bcil). The modifications for CBVw fMRI preprocessing are summarized in Figure 2.

#### 2.4.3 Structural image pre-processing

Structural image processing followed our previous protocol and is described in more detail elsewhere (Autio et al., 2020; Glasser et al., 2013). In brief, the NHP*–*HCP PreFreeSurfer pipeline was used to align structural T1w and T2w images into an anterior-posterior commissural (AC-PC) line using a rigid body transformation, remove non-brain structures, align T2w and T1w images using boundary-based registration (Greve & Fischl, 2009), and correct for signal intensity inhomogeneity. Sphinx-positioned brains, which are often misoriented by the scanner, were corrected using a “CorrectVolumeOrientation.sh.” The removal of non-brain structures and alignment to AC-PC lines were also significantly improved by implementation of “bet4animal” in FSL 6.0.6.6.

FreeSurferNHP pipeline was used to reconstruct the cortical surfaces using FreeSurfer v6.0 (Fischl, 2012). This process included scaling of brain size by a factor of 2 (using “ScaleVolume.sh”), intensity correction, segmentation of the brain into cortex and subcortical structures, reconstruction of the white and pial surfaces, and estimation of cortical folding maps and thickness. The intensity correction was performed using FMRIB’s Automated Segmentation Tool (FAST) (Zhang et al., 2001) and whole brain intensity was scaled by a species-specific factor (80). The white matter segmentation was modified by filling a white matter skeleton to accurately estimate white surface around the blade-like thin white matter particularly in the anterior temporal and occipital lobe. Next, the pial surface was initially estimated by using intensity-normalized T1w image and then modified using the T2w image to exclude dura and blood vessels (Glasser et al., 2013). After this, all the volumes and surfaces were rescaled to the original brain size. The quality of surfaces was visually inspected and, where needed, manual edits were made to the brain, white and ribbon segments (by J.A., T.I., T.H.). The surfaces were then re-evaluated by running a pipeline using the edited segments.

The PostFreeSurfer pipeline transformed anatomical volumes and cortical surfaces into the standard macaque space (Hayashi et al., 2021), performing surface registration using folding information via MSMSulc (Robinson et al., 2018). Surface models and data were resampled to a high-resolution 164k mesh (per hemisphere), as well as lower resolution mesh (10k) for processing functional MRI data.

#### 2.4.4 Quantitative relaxation mapping

The quantitative T_1_ and T_2_* images were brain masked, aligned into an anterior-posterior commissural (AC-PC) line using a rigid body transformation. Cortical surface mapping (in the 10k CIFTI format) was performed with wb_command –volume-to-surface-mapping using the value of enclosing voxel at the white and gray matter surface. The estimated T_1_ median in gray matter (T_1_ = 1.37 s) was used to determine Ernst angle for CBVw fMRI (TR 0.75 s yields FA=55°), whereas estimated T_2_* median in gray matter was used to compare optimal TE for CBVw fMRI (TE=∆T_2_*).

#### 2.4.5 Functional data pre-processing

The CBVw or ferumoxytol-weighted functional MRI processing was adapted from our previous BOLD NHP-HCP fMRI pipeline (Autio et al., 2020; Glasser et al., 2013), with modifications to address unique pre-processing challenges associated with CBVw imaging. Functional images were motion corrected using MCFLIRT (Jenkinson et al., 2002). Because MCFLIRT has a set of hard-coded assumptions of registration resolution, the brain size was scaled by a factor of 2 and rescaled to the original before and after MCFLIRT, respectively. Then images were corrected for geometric B_0_-distortions using spin-echo field maps and TOPUP (Andersson et al., 2003) with fMRI resolution and readout-adapted configuration (b02b0_macaque_fMRI.cnf).

The injection of ferumoxytol contrast agent reduced the signal intensity and contrast between white and gray matter, degrading the alignment of functional images to AC-PC aligned anatomical T1w images. To address this limitation, we implemented a multi-step registration strategy: registration was initialized using the spin-echo field-map magnitude image. This process involved creating a synthetic B_0_-distorted T1w image derived from a B_0_ field map, which facilitated initial alignment. Subsequent registration was refined using the undistorted spin-echo magnitude image. We applied an optimized pial surface approach using “boundary-based registration” (BBR) (Greve & Fischl, 2009) to achieve precise alignment of fMRI to T1w. This optimized method used the contrast between the outer brain boundary and cerebrospinal fluid (CSF), using a brain mask using FSL’s BBR for the initial volume-based registration followed by tuned registration using pial surfaces in FreeSurfer’s BBR. The FSL BBR was performed using T1w contrast (slope = 0.5 in FSL’s BBR) and a brain mask volume created from pial surfaces using the FreeSurferNHP pipeline. FreeSurfer BBR employed an option of using T1w contrast (—t1), and a white and gray matter projection absolute distances of 1.1 and 0.2 mm, respectively, and smaller resolutions in the initial brute force search (—brute1max 2 and —brute1delta 2) to take into account the brain scale of NHP brain. This “pial surface BBR” approach for ferumoxytol fMRI effectively resulted in robust and accurate alignment between fMRI and anatomical images. The registration accuracy was validated by estimating minimum cost function—initial registration with spin-echo (field-map) magnitude images resulted in low minimum cost function values (0.03 ± 0.01 for FSL’s BBR; 0.42 ± 0.10 for FreeSurfer BBR; n = 108 sessions); and single-band gradient-echo registration also yielded low values (0.11 ± 0.02, max = 0.16 for FSL’s BBR; 0.08 ± 0.04, max = 0.25 for FreeSurfer BBR). Finally, functional data were normalized to the grand 4D mean and masked to include gray matter structures.

The cerebral cortical gray matter voxels were mapped to the surface with the partial volume-weighted ribbon-constrained volume-to-surface mapping algorithm, and voxels having large deviations (greater than 0.5 s.d. above mean) from the local (in a 5 mm sigma Gaussian) neighborhood voxels’ coefficient of variation were excluded (Glasser et al., 2013). Data were minimally smoothed at 1.25 mm full-width half-maximum (FWHM) using geodesic Gaussian surface smoothing algorithm with vertex area correction and resampled according to the folding-based registration (MSMSulc) to a standard mesh in which the vertex numbers correspond to neuroanatomically matched locations across subjects. The subcortical gray matter voxels were processed in the volume using 1.25 mm FWHM subcortical parcel-constrained smoothing and resampling. Altogether, these processes transformed the functional data into a standard set of grayordinates (≈10,000 [10k] vertices per hemisphere and ≈8,000 [8k] subcortical voxels) in the Connectivity Informatics Technology Initiative (CIFTI) format (Autio et al., 2020; Glasser et al., 2013).

#### 2.4.6 Group-level functional connectivity

Structured temporal noise, which arises from imaging artifacts such as motion and physiological fluctuations, was identified and removed using a macaque-adapted version of FMRIB’s spatial ICA-based X-noisefier (FIX) (“ICA + FIX”) (Glasser et al., 2018; Griffanti et al., 2014, 2017; Salimi-Khorshidi et al., 2014). To further distinguish between meaningful neural signal components from unstructured noise, we applied principal component analysis (PCA). This allowed us to separate data into two subspaces: (1) a structured subspace, which contains signals that exhibit meaningful spatial and temporal patterns, and (2) an unstructured subspace, which consists of signal components that follow a random statistical distribution (modeled as a Wishart null distribution) and are, therefore, considered noise (Glasser et al., 2016). Unstructured (Gaussian) noise primarily originates from the MRI system itself, including the RF-transmit coil, the sample (head), the gradient coils, the RF-receive coil, and pre-amplifier. In contrast, structured (non-Gaussian) noise results from imaging artifacts such as motion and physiological fluctuations, which exhibit characteristic spatial and temporal patterns. The ICA-FIX cleaned structured subspace was further analyzed at the group-level functional connectivity by correlation of each vertex’s eigenvalues with each other and computing a dense Pearson’s correlation matrix.

Structured temporal noise arising from imaging artifacts (e.g., motion and physiological noise) were manually classified and removed using a macaque-adapted version of FMRIB’s ICA-based X-noisefier (FIX) (“sICA + FIX”) (Glasser et al., 2018; Griffanti et al., 2014, 2017; Salimi-Khorshidi et al., 2014). Principal component analysis (PCA) was applied to segregate data into structured and unstructured sub-spaces (the latter following Wishart null distribution) (Glasser et al., 2016), and the structured subspace was decomposed into statistically independent components using spatial ICA. The resulting components were manually classified as “signal” or “noise”, based on their spatial distribution and temporal properties (Griffanti et al., 2014).

The CBVw FIX classifier was then trained on this manual classification (N = 3; total trials = 27; total data points = 84,132) and its performance was characterized by true positive rate and true negative rate. Classifier performance was evaluated using leave-one-out accuracy testing across a range of thresholds. The training file will be included in the coming FSL release.

Information on the different categories of CBVw fMRI fluctuations was evaluated using the HCP’s RestingStateStats adapted for macaque monkeys (Autio et al., 2020; Marcus et al., 2013). In brief, RestingStateStats script decomposes total CIFTI fMRI timeseries variance (prior to any preprocessing) into six categories: low-frequency noise (by high-pass filter), motion, artifacts, and nuisance signals (by FIX classification), unstructured noise (by PCA, see above), neural CBVw fluctuations (by FIX classification), and FIX-cleaned mean grayordinate timeseries (MGT). The fractional contribution of each category was calculated by dividing by the total fMRI variance in the grayordinate CIFTI format, which contains cortical gray matter in surface GIFTI format and subcortical structures in volume NIFTI format. The CNR was determined as the ratio of neural variance to unstructured noise variance.

To investigate within- and between-subjects FC reproducibility, the cleaned fMRI timeseries from each imaging session were parcellated using the M132 atlas (Markov et al., 2014). A Pearson’s correlation matrix was calculated to represent FC for each session. The lower triangular portion of each session’s matrix was compared across sessions using Spearman’s rank correlation coefficient.

Subject identification accuracy was quantified by identifying the session pair with the highest Spearman’s rank coefficient. A score of 1 was assigned if the highest correlation corresponded to the same subject, and a score of 0 was assigned otherwise. Scores were averaged across all sessions to compute overall identification accuracy. Additional subject identification analysis was performed using noise-dominant timeseries, in which neural components were regressed out and the noise components were kept in the timeseries.

To explore the functional organization of the macaque brain using group-level FC analyses, we first normalized each session’s dense timeseries for variance to account for potential differences in ferumoxytol concentrations across sessions and subjects. We then re-constructed the group data using a Wishart null distribution threshold to remove redundant unstructured noise in the eigenvalue subspace (Glasser et al., 2016). The reconstructed data were subsequently used to compute a Pearson’s correlation matrix and examine FC across the macaque gray matter.

## 3 Results

### 3.1 Coil performance

**Fig. 3.**
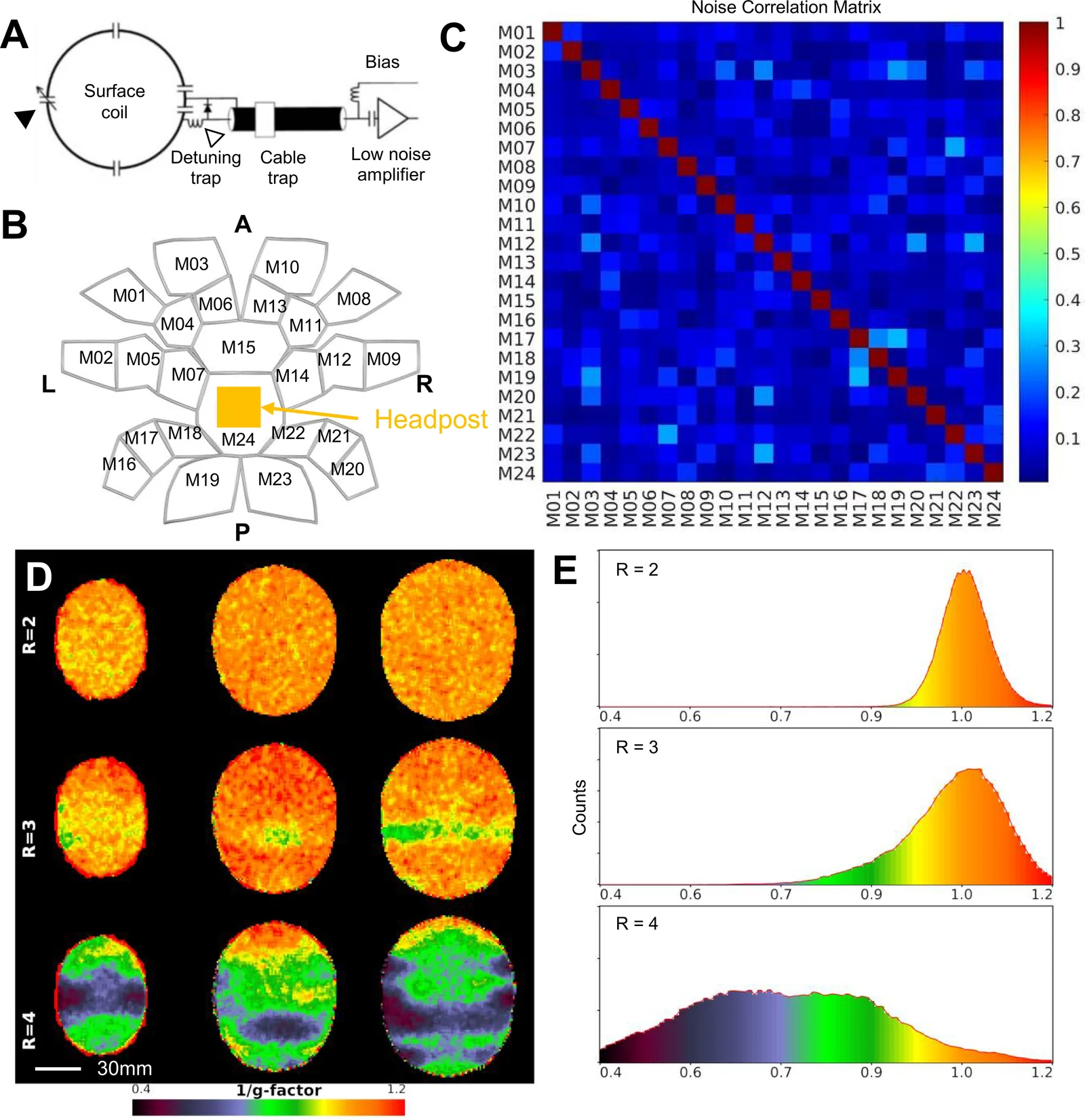
Assessment of the 24-channel coil’s parallel imaging performance. (A) Circuit diagram. White arrow head: an inductor of detuning trap; black arrow head: trimmer capacitor. (B) Flattened map of the coil element arrangement. The orange arrow indicates the opening in the phased array designed to accommodate the rigid head attachment. (C) Noise correlation matrix. (D) Inverse geometry (1/g)-factor maps obtained using gradient-echo imaging with in-plane acceleration using GeneRalized Autocalibrating Partially Parallel Acquisitions (GRAPPA) at different reduction factors. (E) 1/g-factor histograms.

Table 1 provides an overview of the quality assurance measures conducted on the newly developed 24-channel phased-array coil for awake macaque imaging. Our assessment showed a maximum active detuning of -20 dB, with an average S21 of -28 ± 6 dB across 54 adjacent coil element pairs, reflecting the combined effects of geometric overlapping and preamplifier decoupling, and low noise correlation (0.075 ± 0.052). For comparison, this noise correlation is lower than that reported for Siemens’s 32-channel human head receive coil (0.117; Wiggins et al., 2006), and previously published macaque coils (0.22, Janssens et al., 2013; 0.13, Gilbert et al., 2016; 0.13, Quan et al., 2020), and is comparable with our previous macaque coil design (0.084, Autio et al., 2020). In-plane acceleration using GRAPPA R=2 resulted in an inverse g-factor marginally above unity, followed by slight reduction in R=3, and a notable drop at R=4, reflecting the upper limit of the coil’s parallel imaging capability (Fig. 3D, E).

**Table 1.** Quality assurance of awake (top row) and anesthetized (bottom row) macaque 24-channel phased-array receive coils.

Coil	Q-ratio	Maximum active detuning (dB)	Noise corr.	1/g	B_1_ +	Reference
Awake	215/75 = 2.9	-20	0.08 ± 0.05	1.14 ± 0.11	89.0° ± 4.5	Current study
Anesthetized	215/75 = 2.9	-20	0.08 ± 0.04	1.03 ± 0.07	86.6° ± 2.3	Autio et al. (2020)

Q-ratio is the unloaded to loaded ratio of the individual coil elements. Maximum active detuning indicates detuning performance efficiency when measured detuning ON and OFF. Noise correlation indicates average noise correlation between the coil elements (mean ± std). Inverse geometry (1/g) factor above unity indicates a small noise cancellation during in-plane parallel image reconstruction using a reduction factor R = 2. Relatively homogeneous flip-angle distribution over cortical surface indicates that the coil did not interfere with B_1_-transmission.

**Fig. 4.**
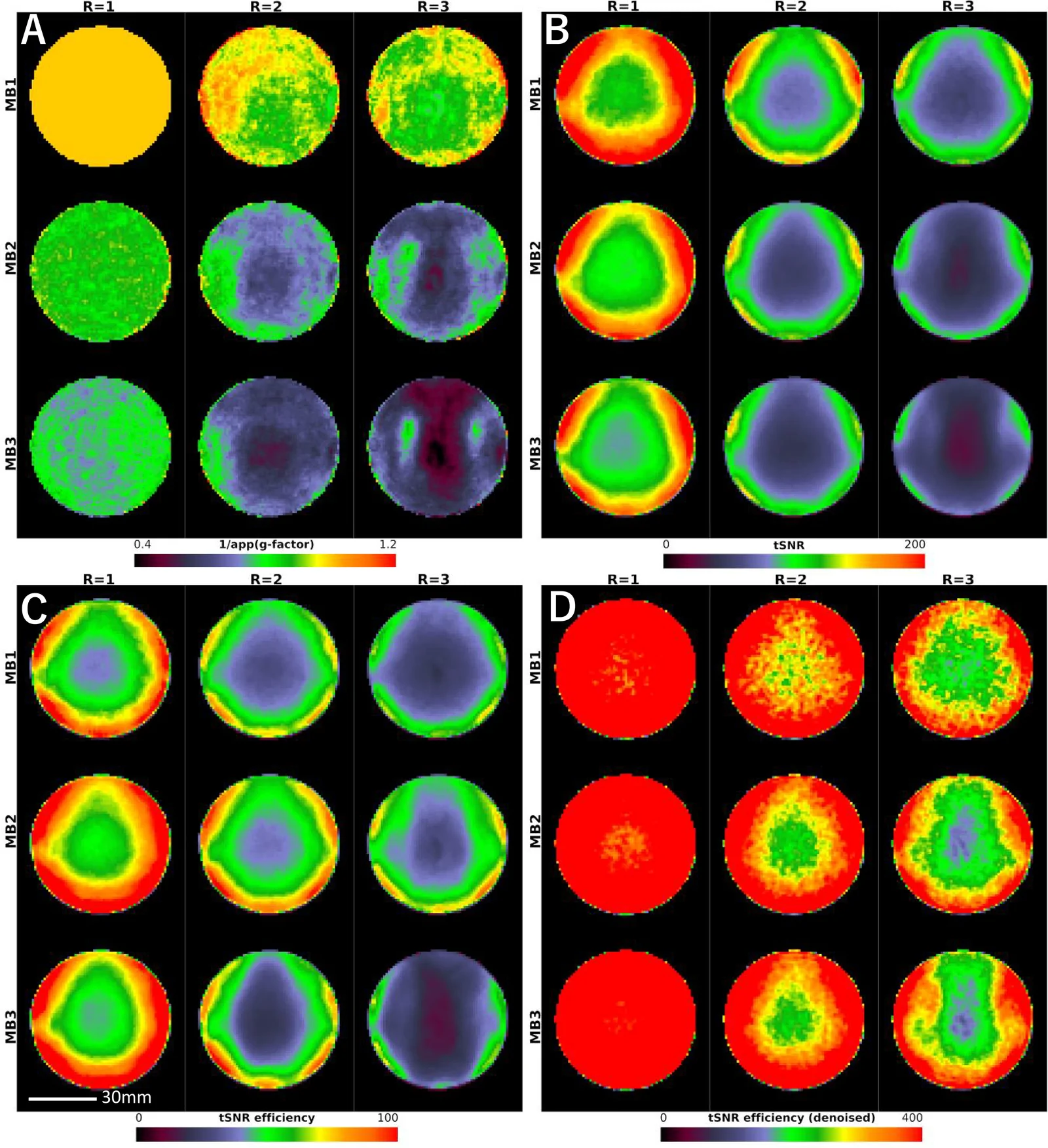
Evaluation of in-plane Generalized Autocalibrating Partially Parallel Acquisitions (GRAPPA) and out-of-plane multiband (MB) acceleration effects on temporal signal characteristics. (A) Inverse geometry-factor (1/g) maps as a function of GRAPPA reduction (R) and MB acceleration factors. (B) Temporal signal-to-noise ratio (tSNR) per volume across varying R and MB combinations. (C) tSNR efficiency per unit time, calculated as tSNR normalized by the square root of the repetition time. (D) Post-ICA tSNR efficiency maps show that combined MB and GRAPPA acceleration preserves signal quality in peripheral regions (approximately corresponding to the cerebral cortex), with moderate signal attenuation in central brain regions.

We evaluated the coil’s parallel imaging performance for MB acceleration and its combination with GRAPPA using EPI and a phantom. MB acceleration reduced the apparent g-factor (Fig. 4A), potentially due to slice cross-talk or signal leakage (Xu et al., 2013). As expected, combining MB with GRAPPA led to further g-factor–related signal degradation. Temporal SNR (tSNR) without acceleration primarily reflected the effective B_1_-distribution and the proximity to the coil elements (Fig. 4B). With increasing acceleration, tSNR decreased, mainly due to incomplete T_1_ recovery and in part due to increased noise via the acceleration.

To account for differences in acquisition speed, we computed tSNR efficiency by normalizing tSNR by the square root of TR (Fig. 4C) (Boyacioğlu et al., 2015). TSNR efficiency showed a mild decline with MB acceleration but a more pronounced reduction with higher in-plane GRAPPA factors. After ICA denoising (Fig. 4D), the MB3 × GRAPPA2 combination provided a favorable balance between speed and signal quality, yielding robust performance in peripheral phantom regions—approximating cortical locations—though some central signal degradation persisted.

**Fig. 5.**
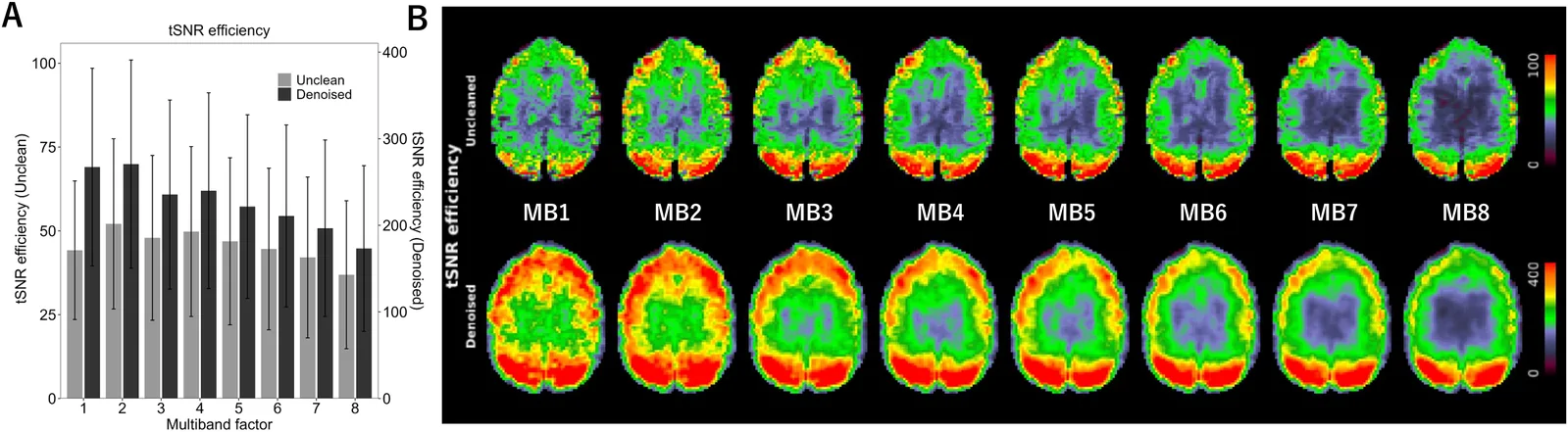
In vivo multiband performance evaluation. (A) Multiband performance evaluation assessed using the temporal signal-to-noise ratio (tSNR) efficiency that is scaled by square root of the TR across different multiband factors. (B) Uncleaned (top row) and ICA-cleaned (bottom row) tSNR efficiency.

In vivo, we also evaluated the coil’s performance for multiband acceleration and observed an increase in tSNR (scaled by the square root of timepoints) at moderate acceleration factors (2–4) followed by a gradual decline (Fig. 5), along with notable cross-slice artifacts (data not shown). ICA denoising improved tSNR efficiency across the brain (Fig. 5A, B), although inferior regions retained lower tSNR due to their greater distance from the receive elements.

These quality assurance measures closely resembled those obtained with our previous 24-channel phased-array coil tailored for imaging anesthetized monkeys (Supplementary Figs. S2, S3) (Autio et al., 2020), with two key differences: slightly elevated variation in the B_1_+ transmission field attributable to the coil’s positioning 10 cm above the B_0_ isocenter in the awake configuration, slightly better MB and slightly worse GRAPPA g-factor performances, likely reflecting differences in number of elements along the slice and phase-encoding directions (Supplementary Fig. S3A, B). The coils showed comparable tSNR, tSNR efficiency, and ICA denoise tSNR efficiency across combined GRAPPA + MB acceleration schemes, supporting efficient imaging at higher acceleration factors (Fig. 4B, C, D; Supplementary Figs. S2, S3). Collectively, these quality assurance results indicate robust parallel imaging capabilities, essential for accelerated CBVw fMRI in awake macaque monkeys.

**Fig. 6.**
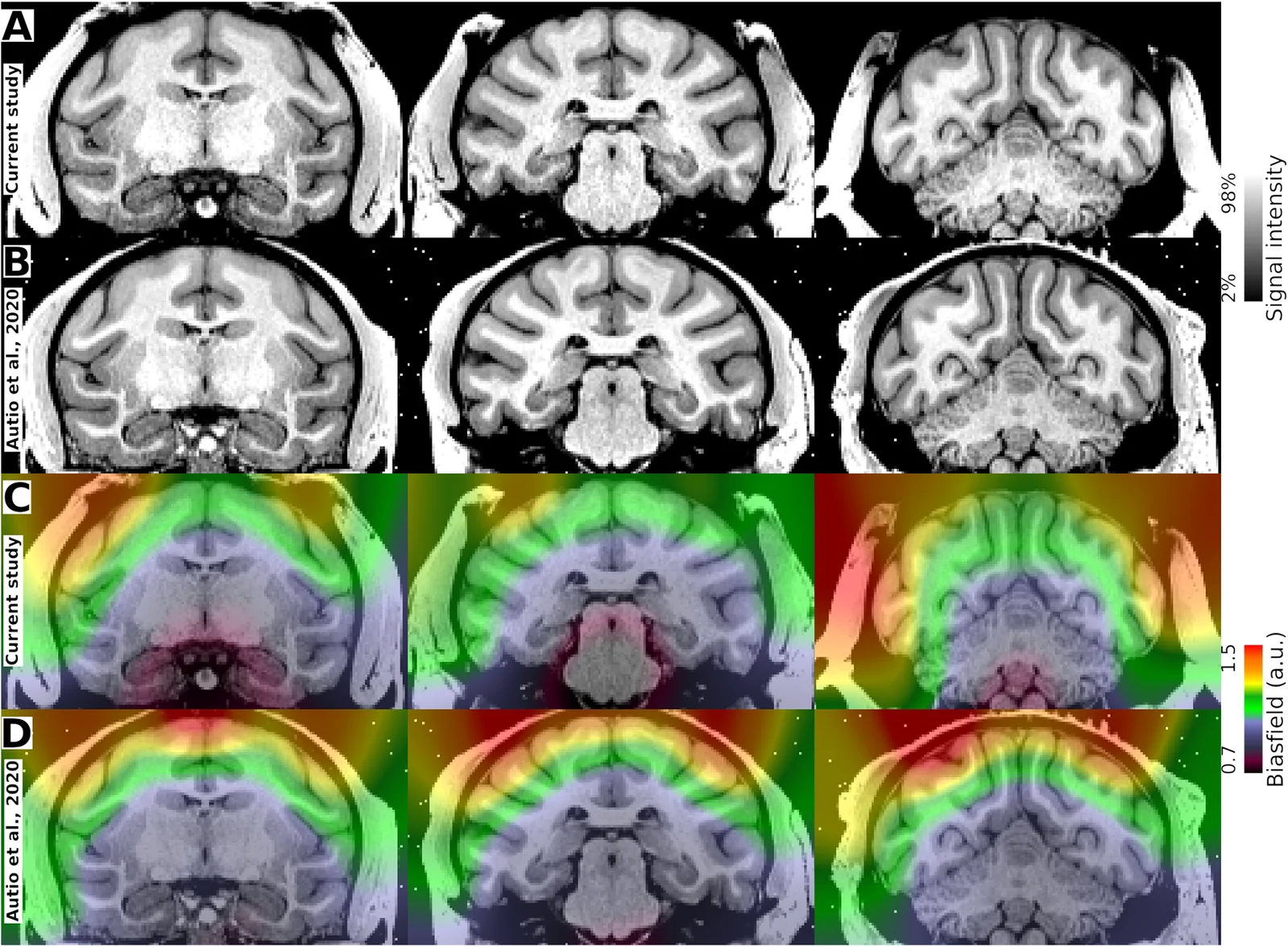
Example of structural image quality assessment. Structural T1w images acquired using a phased-array coil designed for (A) awake (current study) and (B) anesthetized monkeys (Autio et al., 2020). The images were obtained in the same animal before (B) and after headpost placement (A). The image resolution is 0.5 mm isotropic, GRAPPA 2, and acquisition time 17 min. The red and yellow lines indicate pial and white matter surfaces, respectively. (C, D) B_1_ biasfield estimated from T1w and T2w images. Note that the top of the brain exhibits different biasfield primarily due to differences in coil configuration and the absence of extra-brain tissues in animals following headpost surgery.

Representative structural brain images acquired with parallel imaging acceleration are shown in Figure 6. The image quality is comparable with that obtained with our previous coil for anesthetized animals (Fig. 6A, B), although the newly developed coil exhibits slightly lower SNR. Some signal dropout is inevitable due to the phased-array opening designed to accommodate headpost fixation. However, in-vivo scanning is complicated by the absence of extra-brain tissues following head-post surgery, which possibly alters RF penetration. Indeed, the signal loss, as well as the B_1_ biasfield, near the top of the brain was relatively attenuated following surgery, relative to that scanned with our previous coil in the same animal before surgery (Fig. 6C, D). Despite the B_1_ biasfield variations of approximately 50% throughout the cortex, SNR remains sufficient to enable robust image segmentation and accurate delineation of white matter and pial surfaces (Fig. 6A, red and yellow lines).

### 3.2 Contrast agent dose optimization

**Fig. 7.**
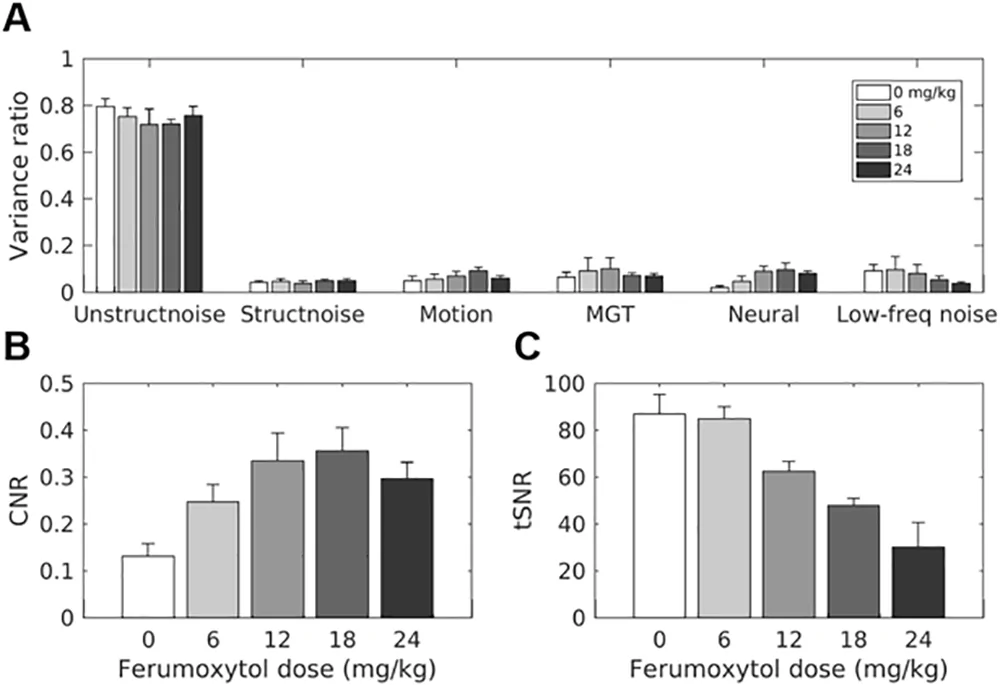
Determining optimal ferumoxytol contrast agent dose for accelerated cerebral blood volume-weighted (CBVw) resting-state fMRI. (A) Variance categories, (B) contrast-to-noise ratio (CNR), and (C) temporal signal-to-noise ratio (tSNR) at varying ferumoxytol doses. Data represent mean, with error bars indicating the standard deviation across image runs and subjects (n = 2 × 3 = 6). Data were analyzed using the Human Connectome Project’s RestingStateStats (Marcus et al., 2013). Abbreviations: Low-freq noise, low-frequency noise (by high-pass filter); StructNoise, structured noise (by FIX classification); Signal (by FIX classification); UnStruct Noise, unstructured noise (by PCA), MGT FIX-cleaned mean grayordinate timeseries.

Pilot experiments were conducted to evaluate the impact of ferumoxytol contrast agent concentration (0, 6, 12, 18, and 24 mg/kg) on resting-state fMRI fluctuations in anesthetized macaques (N=3). The objective was to quantify the relative variance of unstructured noise, low-frequency filtered noise, structured noise, neural signal fluctuations, and FIX-denoised mean global timeseries (MGT) (Autio et al., 2020; Marcus et al., 2013). The relative neural signal variance exhibited a dose-dependent response to ferumoxytol contrast agent, peaking at concentrations between 12 and 18 mg/kg, and declining at 24 mg/kg (Fig. 7A, B). These results align with classical single-tissue-compartment CNR optimization principles, where the TE (15 ms) closely matches the average T_2_* of gray matter at approximately 16 ms and 14 ms for doses of 12 mg/kg and 18 mg/kg, respectively (see Appendices, Data Acquisition Strategy) (Boxerman et al., 1995; Mandeville et al., 2004). Considering the marginal difference in CNR between the 12 and 18 mg/kg doses and the sufficient tSNR across the gray matter (Fig. 7C), the 12 mg/kg dose was chosen for subsequent awake CBV fMRI experiments.

It is also noteworthy that tSNR, a commonly used metric to assess functional MRI data quality in terms of signal detectability, decreases progressively with increasing concentrations of ferumoxytol contrast agent (Fig. 7C). This occurs because ferumoxytol injection reduces overall SNR while amplifying neural signal fluctuations (Fig. 7A, B), both leading to a weak tSNR, which is defined as tSNR = signal fluctuation/mean MR signal. For this reason, we consciously refrained from relying on tSNR maps, and instead focused on quantifying neural signal content, as identified using spatial ICA.

### 3.3 ICA-FIX cleanup and reproducibility of functional connectivity

To evaluate the fMRI data quality, we categorized fMRI timeseries (totaling ≈1,000 min and ≈84,132 volumes) into unstructured noise (via PCA), structured noise (via spatial ICA) motion, mean grayordinate timeseries, neural signal (via ICA), and low-frequency noise using the FIX (Griffanti et al., 2014, 2017; Salimi-Khorshidi et al., 2014) using a modified HCP resting-state analysis pipeline for macaque monkeys (Autio et al., 2020; Marcus et al., 2013). The average spatial distribution of each category is shown in Figure 6A.

**Fig. 8.**
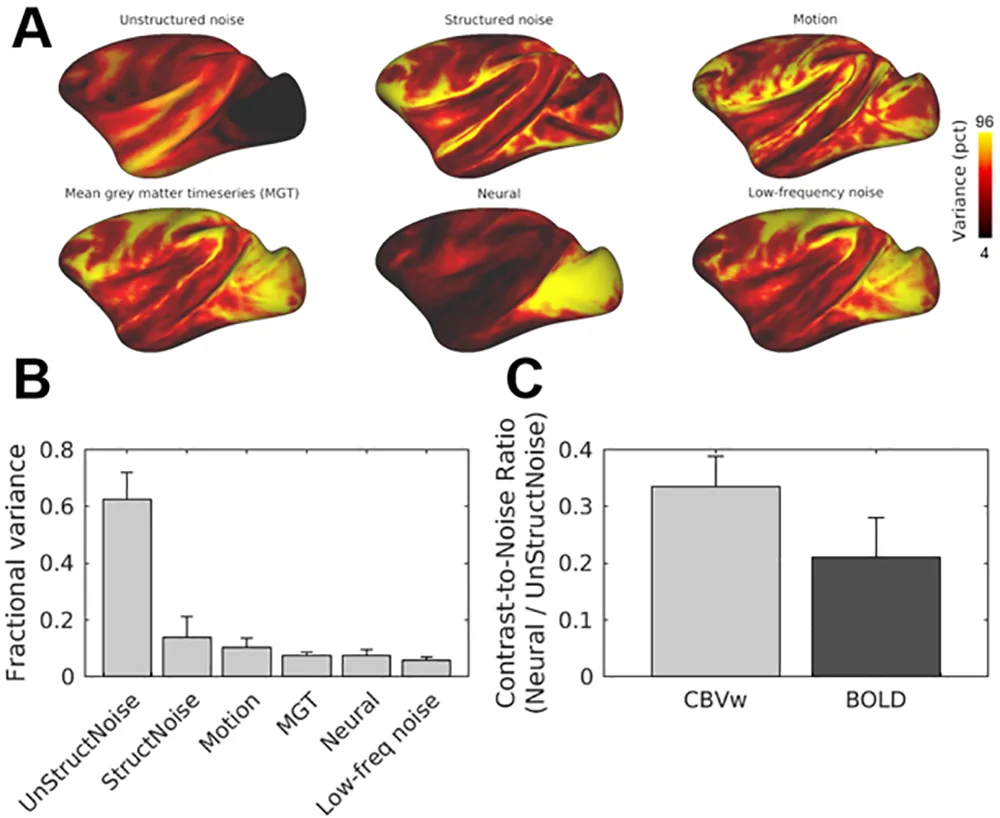
Classification of Noise and Neural Variance in Resting-State fMRI Timeseries. (A) Averaged resting-state categories displayed on a cortical flat map (N=3, total n=27 sessions). (B) Summary of average fractional variances across the categorized signals. (C) Comparison of contrast-to-noise ratio (CNR) between cerebral awake blood volume-weighted (CBVw) and anesthetized blood oxygen level-dependent (BOLD) resting-state fMRI, calculated as the ratio of neural signal variance to unstructured noise variance ratio. Fractional variances for each category were estimated using the HCP RestingStateStats pipeline (Autio et al., 2020; Marcus et al., 2013). Abbreviations: UnStructNoise: unstructured noise; StructNoise: structured noise; MGT: mean gray matter timeseries.

On average, unstructured noise accounted for the largest portion of fMRI signal variance (60%) (Fig. 8B). Unstructured noise was higher in brain regions more distant to coil elements, as expected. In contrast, neural signals were strongest in the visual cortex, likely due to its proximity to coil elements as well as its high baseline vascular volume and neuron density (Autio et al., 2025; Collins et al., 2010). Structured noise, motion, and low-frequency noise in total contributed to a 4-fold larger amount of variance than neural signals (30% vs 7%). After removing these artifacts, the resulting CNR, defined as the ratio between neural signal and unstructured noise variances, was significantly larger than in anesthetized BOLD fMRI (p < 10^-7^, Fig. 8C) (Autio et al., 2020).

**Fig. 9.**
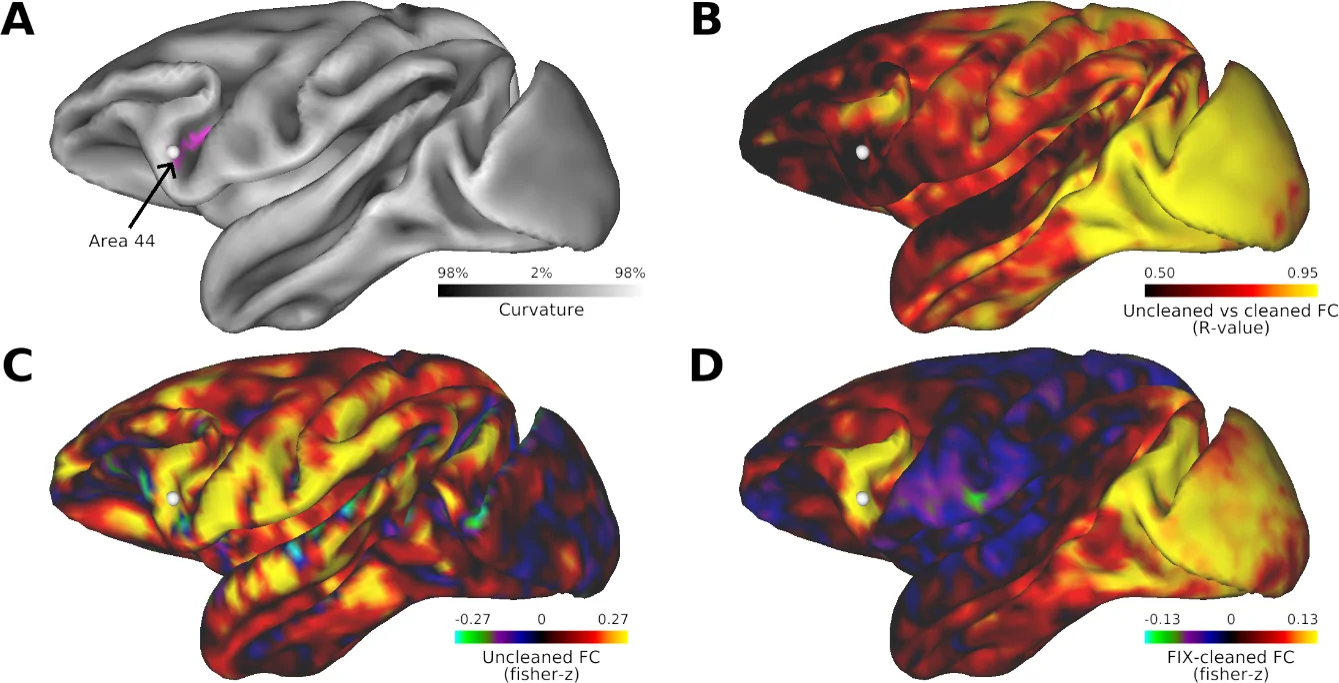
Accelerated Ferumoxytol-weighted fMRI in Combination ICA-FIX Artifact Cleanup Improves Functional Connectivity Estimation in Awake Macaque Cerebral Cortex. (A) Seed location at area 44 (white circle) as indicated by M132-atlas (magenta). (B) Spatial correlation patterns between FIX-uncleaned and FIX-cleaned functional connectivity (FC) (N=1; imaging sessions=9). Note that the correlation between FIX-uncleaned and cleaned FC varied between 0.53 and 0.95 (5th and 95th percentile), highlighting the importance of data-driven ICA de-noising in ferumoxytol-weighted fMRI. Exemplar seed FC from area 45 (C) prior to and (D) after FIX-cleanup.

An exemplar FC map seeded from bottom of the anterior sulcus (Fig. 9A) shows pronounced differences before and after ICA-FIX cleanup (Fig. 9C, D). Correlating all seed-based FCs before and after FIX cleanup reveals that FC is more profoundly improved by FIX in the anterior regions of the brain, with minimal impact in the posterior visual system (Fig. 9B). This anterior-posterior gradient can be partially attributed to the headpost fixation at the posterior part of the skull (Fig. 1), which likely results in the anterior regions of the brain being more susceptible to subtle motion artifacts than the posterior regions (Fig. 8A; see motion panel).

Manually classified sICA components (on average 87 ± 53 noise and 18 ± 7 signal components per fMRI session) were used to train an automatic classifier (python FIX; pyFIX) for denoising ferumoxytol-weighted fMRI data. The classifier demonstrated excellent performance (Table 2), with leave-one-out testing showing a perfect true positive rate of 100% ± 0%. True negative rate ranged from 85% to 100%, depending on the threshold level. Consequently, a threshold of 40 was selected for automatic classification, resulting in a mean true positive rate and true positive rate of 100%.

**Table 2.** FIX classification accuracy test by leave-one-out (LOO) in 27 imaging sessions of awake macaque data.

FIX threshold	1	2	5	10	20	30	40	50
TPR (mean)	1.00	1.00	1.00	1.00	1.00	1.00	1.00	1.00
TNR (mean)	0.86	90.00	0.95	0.97	0.98	0.99	1.00	1.00
TPR (median)	1.00	1.00	1.00	1.00	1.00	1.00	1.00	1.00
TNR (median)	0.85	0.92	0.97	0.98	0.99	1.00	1.00	1.00

Abbreviations: TPR: true positive rate of signal components; TNR: true negative rate of true artifact components.

Next, we investigated the dependence of FC reproducibility on scan duration using M132 atlas-parcellated correlation matrices (Birn et al., 2013; Laumann et al., 2015). We analyzed data from three awake macaques, each with nine imaging sessions (totaling ≈1,000 min and ≈84,132 volumes) and from five anesthetized macaques each with two imaging sessions (totaling 1,020 min and 81,920 volumes). We generated parcel timeseries and performed within-scan reproducibility analysis by systematically varying the number of fMRI volumes (Supplementary Fig. S4A). Our analysis revealed a reproducibility of ρ = 0.94 ± 0.03 (Spearman rank ± standard deviation) in awake macaques and ρ = 0.94 ± 0.04 in anesthetized macaques (Supplementary Fig. S4B). Notably lower reproducibility was observed in subcortical structures in anesthetized macaques (data not shown), suggesting an impact of anesthesia on the consistency of connectivity patterns, which could be further explored to understand differences in brain dynamics between these states.

**Fig. 10.**
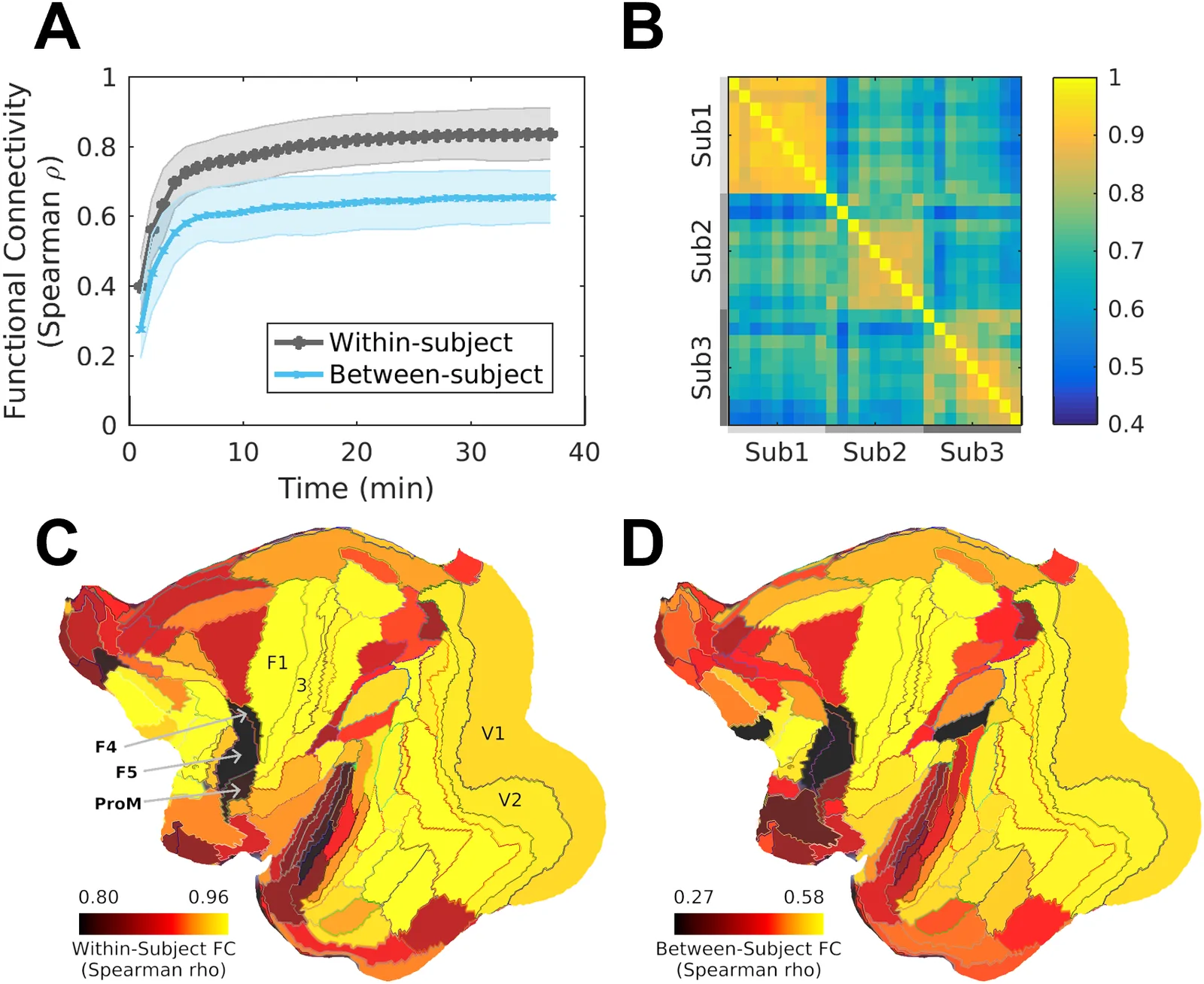
Within- and Between-Subject Reproducibility of Resting-State Functional Connectivity. (A) Within-subject and between-subject functional connectivity (FC), parcellated using the M132 atlas, plotted as a function of scan duration. The continuous line represents the mean Spearman’s rank correlation, while the shaded error bars represent the standard deviation. (B) Reproducibility of within-subject and between-subject FC. (C) M132-parcellated FC matrices from three awake macaques across nine imaging sessions each. (D) Parcel-wise FC reproducibility within subjects (left) and between subjects (right) displayed on a cortical flat map. A total of 182 parcels were analyzed, yielding 16,380 parcellated FC values across both hemispheres of the cerebral cortex.

The within-subject reproducibility of the M132 atlas-parcellated FC matrix was excellent ρ = 0.84 ± 0.08 (Fig. 10A, B). The between-subject FC matrix correlation was moderate 0.66 ± 0.08, and comparable with those reported in humans (Koike et al., 2021). The lower between-subject correlation indicates that each subject’s FC matrix is distinct. Notably, each individual could be accurately distinguished across all imaging sessions using any random pair of scans, achieving 94% subject identification accuracy. The scans that were not correctly identified had large, persistent motion artifacts (data not shown). This furthers the idea that FC serves as a reliable neural fingerprint to identify individual humans (Finn et al., 2015) also in macaques based on their distinctive neural activity patterns.

To further investigate individual differences, we examined noise patterns (e.g., noise-classified ICAs) that might also be subject specific due to variations in large vessel locations, brain shape, and idiosyncratic movements. The within-subject reproducibility of the M132 atlas-parcellated noise matrix was good (ρ = 0.79 ± 0.07), while the between-subject correlation was moderate (ρ = 0.56 ± 0.07) (Supplementary Fig. S5), indicating that each subject’s noise correlation matrix is distinct. These noise patterns alone enabled high identification accuracy across subjects, reaching 98%. This demonstrates that noise-dominant correlation patterns can also serve as reliable identifiers of individual macaques.

### 3.4 Group-level functional connectivity

**Fig. 11.**
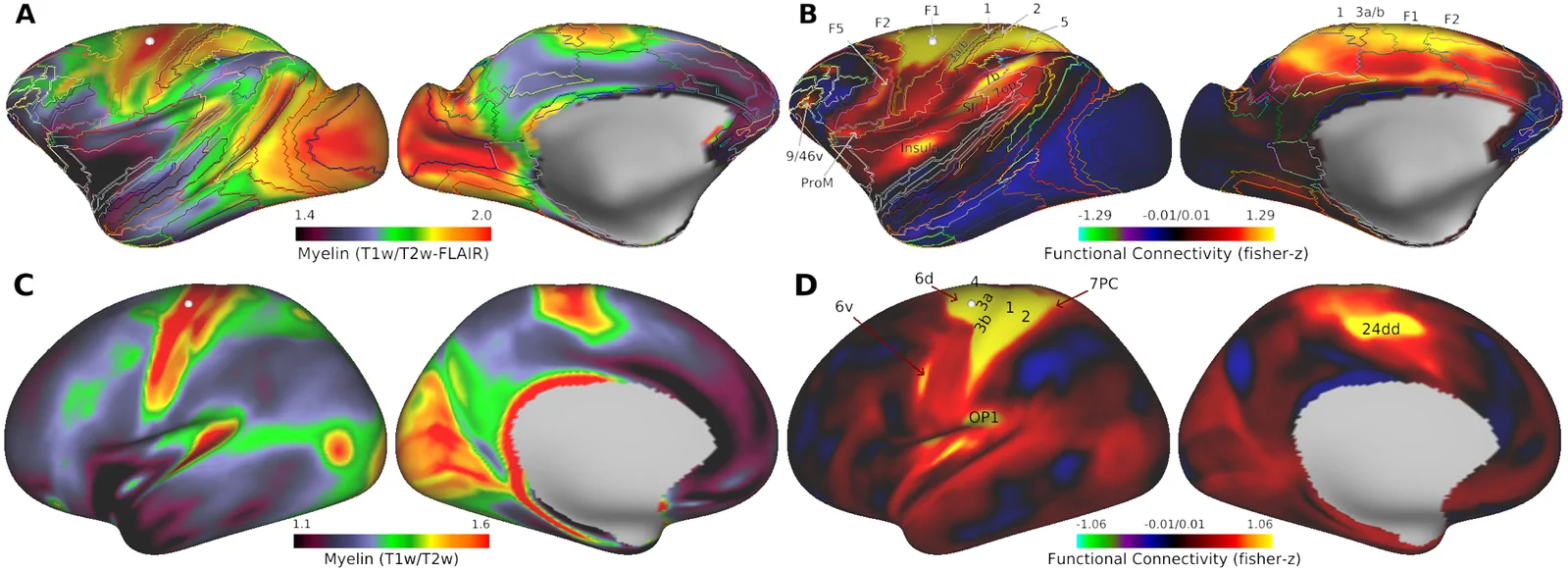
Comparison of Group-Level Resting-State Functional Connectivity in Awake Macaque Monkeys and Humans. (A) Macaque myelin map (Autio et al., 2024). The lines indicate M132-atlas area boundaries (Markov et al., 2014). (B) Resting-state functional connectivity (FC; N=3; total n=27) seeded from the primary motor cortex region of upper-limb presentation. (C, D) Human myelin map and temporal-ICA cleaned FC (N=1200). Notably, the upper-limb presentation appears to cover a larger fraction of the cerebral cortex in macaques than in humans. Abbreviations: M1: primary motor cortex; OP1: opercular area 1; SII: secondary somatosensory area; 1: area 1; 2: area 2; 3a: primary sensory area 3a; 3b: primary sensory area 3b; 5: area 5; 6a: area 6 anterior; 6d: area 6 dorsal; 6dr: area 6 dorsorostral; 6v: area 6 ventral; 7a: area 7a; 7b: area 7b; 7op: area 7 opercular cortex; 7PC: area 7 of parietal cortex; 9/46d: area 9/46 dorsal; 9/46v: area 9/46v ventral; 24dd: area 24d dorsal.

Having established excellent data reproducibility at the individual subject level, we next aimed to explore functional organization of the macaque brain using group-level connectivity analyses. For this objective, we re-constructed the group data using a Wishart null distribution threshold for removing redundant unstructured noise (Glasser et al., 2016). An example of seed-based FC from the upper-limb motor cortex (M1; Fig. 11A) is shown in Figure 11B.

**Fig. 12.**
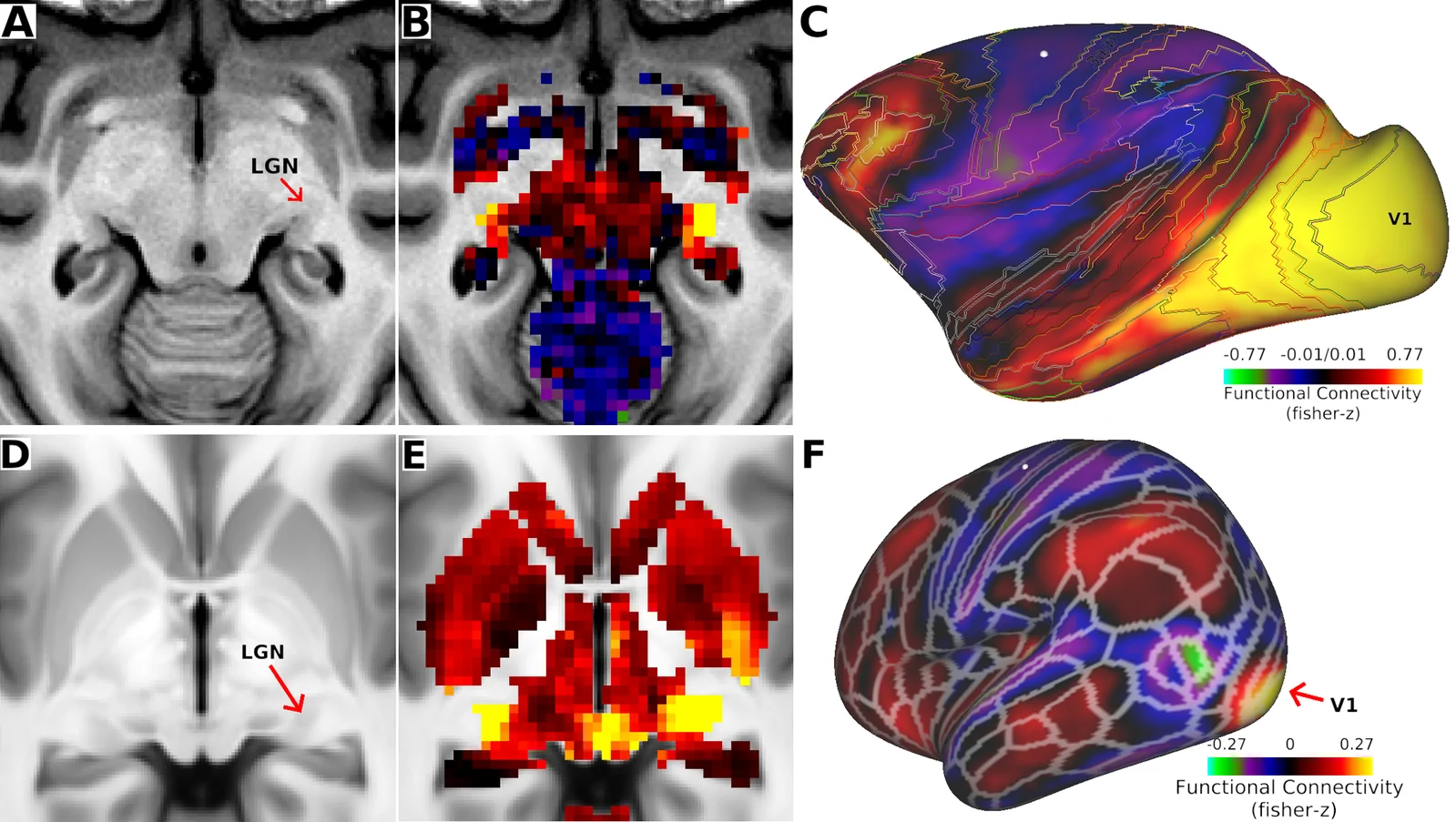
Exemplar Subcortico-Cortical Resting-State Functional Connectivity in Awake Macaque Monkeys and Humans. (A) Seed location in the lateral geniculate nucleus (LGN), (B) subcortical, and (C) cortical functional connectivity patterns. (D, E, F) Human temporal-ICA cleaned FC (N=1200). Cerebral cortex is rotated to display V1. Abbreviations: V1: primary visual area.

Seeding from a similar location in human HCP data reveals a strikingly similar spatial pattern when compared with macaques (Fig. 11C, D). The upper limb network in macaques appears to occupy a substantially larger proportion of the cerebral cortex compared with humans. Likewise, seeding from the lateral geniculate nucleus reveals comparable subcortico-cortical FC patterns, though their relative extent varies with respect to the cerebral cortex (Fig. 12A-F). Together, these results demonstrate the effectiveness of the newly developed 24-channel RF receive coil, ferumoxytol-weighted fMRI, and modified NHP–HCP pipelines in mapping the functional organization of the macaque gray matter.

## 4 Discussion

Here, we have presented a neuroimaging approach for awake macaque monkeys using a combination of a custom-made headpost-compatible 24-channel receive RF coil, three-by-two accelerated (out-of-plane MB and in-plane GRAPPA) ferumoxytol-weighted fMRI, and modified HCP–NHP preprocessing and analysis pipelines. Importantly, this approach facilitates robust and reproducible functional investigations, effectively addressing the reproducibility challenges associated with NHP neuroimaging (Autio et al., 2021). The resources developed, including the 24-channel macaque coil (via Rogue Research, Montreal, Canada https://www.rogue-research.com/; produced by Takashima Seisakusho Co. Ltd. Tokyo, Japan) along with the data acquisition protocols (https://brainminds-beyond.riken.jp/hcp-nhp-protocol) and NHP–HCP CBVw fMRI analysis pipelines (https://github.com/Washington-University/HCPpipelines), are publicly available. Raw and minimally preprocessed awake ferumoxytol-weighted resting-state fMRI data will also be shared in an upcoming publication, as a part of the NHP–HCP neuroimaging and neuroanatomy project (Hayashi et al., 2021), enabling the broader scientific community can leverage these developments for further research.

The newly developed coil additionally provides high-quality structural images important for accurate segmentation and robust cortical surface generation (Fig. 6A), a fundamental requirement for surface-based fMRI analysis. Surface mapping is becoming increasingly important for integrating functional, histological, receptor, genetic, and transcriptomic datasets (Chen et al., 2023; Froudist-Walsh et al., 2023; Hayashi et al., 2021).

Although the benefits of contrast agents have been well documented in task-based fMRI studies (Leite et al., 2002; Mandeville et al., 2004; Vanduffel et al., 2001; Zhao et al., 2006), the majority of macaque resting-state fMRI research at 3T continues to rely on BOLD contrast (Autio et al., 2021; Milham et al., 2018). However, the relative neural BOLD signal variance in anesthetized macaque monkeys at 3T is modest (≈0.1–3%) (Autio et al., 2021; Milham et al., 2018), even when using multiband accelerated imaging sequences (≈4%) (Autio et al., 2020). Ferumoxytol contrast agents provide means to amplify the resting-state fMRI signal fluctuations, increasing the relative signal variance to approximately 7% (Fig. 7A, 6B). The observed 2.5-fold CNR increase for resting-state (from 0.13 ± 0.03 to 0.34 ± 0.6; Fig. 7B) is close to the previously reported 3-fold CNR increase achieved using a block task paradigm (Leite et al., 2002; Vanduffel et al., 2001). However, relative to anesthetized BOLD (0.34 ± 0.06 vs. 0.21 ± 0.07; Fig. 8C), the improvement is more modest.

Despite the improvement in CNR and the rigid head coil attachment, functional timeseries in awake macaques contain a large amount of spatially time-varying artifacts (e.g., subtle motion and respiration) that must be removed from the data to obtain neurobiologically sensible FC profiles (Fig. 8, Fig. 9) (Autio et al., 2021; Griffanti et al., 2014; Power et al., 2012). Indeed, motion and structured artifacts accounted for ≈24% of the variance in awake macaque CBVw resting-state fMRI, which together represent about threefold more variance than neural signals (Fig. 8B). Comparatively, in humans, these artifacts are even more pronounced, contributing approximately seven times more variance than neural signals (Marcus et al., 2013). We used sICA-FIX to remove these motion and structured noise artifacts from the data thereby improving seed-based FC estimations (Griffanti et al., 2014). For instance, denoising had a profound effect on CBVw resting-state FC between MT and FEF (Fig. 9) (Markov et al., 2014). These results are in line with our preliminary report that have demonstrated that FIX-ICA denoising improves the comparison between quantitative retrograde tracer and FC in anesthetized macaques (Hayashi et al., 2021; Markov et al., 2014; Van Essen et al., 2019).

One known limitation of the sICA-FIX clean-up, however, is that it does not effectively remove global artifacts (Glasser et al., 2018). This is a potential concern in CBVw fMRI, where the relative global gray matter timeseries variance was notably high before ICA-FIX cleanup (≈7.4%). This global variance may reflect variations in global neural activity (Schölvinck et al., 2010), respiration, and washout of the ferumoxytol contrast agent. In contrast, anesthetized macaque BOLD fMRI experiments, where respiration is externally controlled by a ventilation machine (at ≈0.2 Hz), the global variance is merely 0.2% (Autio et al., 2020, 2021). This raises questions whether anesthesia has a strong suppressive effect on global neural activity or merely reduces variability in respiration. To disentangle these physiological processes in the frequency domain, the temporal sampling rate (e.g., TR) should be increased to above the critical sampling frequency (> 2 × frequency of physiological noise). Unfortunately, temporal resolution used herein (TR = 0.7 s = 1.4 Hz) is insufficient to properly sample the cardiac cycle in awake macaques. Consequently, physiological noise from respiration (≈0.2 Hz), cardiac cycles (≈1-2 Hz), and scanner-related helium-pump artifacts (Kiviniemi et al., 2016) may be aliased into and overlap with slower gradient heating-based signal drift, vascular tone, and neuronal processes (Uchiyama et al., 2007) (mainly <0.1 Hz) in the frequency domain. Harmonizing temporal resolution across species could benefit from adjusting the sampling rates according to the animal’s physiology, albeit this is impractical in smaller species with progressively faster respiration (e.g. ≈0.2, 0.2, 1.3, and 1.8 Hz in human, macaque, marmoset, and rat, respectively) and cardiac cycles (≈1, 2, 5, and 7 Hz, respectively) (Baran et al., 2020; Bishop et al., 2022). To address these challenges, our approach is to acquire a large number of fMRI volumes and to selectively remove the remaining respiration- and ferumoxytol washout-related global artifacts from global neural activity at the group level using temporal ICA (Glasser et al., 2018).

Another important consideration is the variability of FC across subjects within each species (Xu et al., 2019), which might reflect species-specific behavioral traits (Finn et al., 2015; Yokoyama et al., 2021). However, such investigations necessitate careful estimation of measurement biases. Here, we demonstrated that within-scan, within-subject, and between-subjects FC reproducibility are similar between macaques and humans (Koike et al., 2021; Laumann et al., 2015; Murphy et al., 2007). Although the awake macaque population size used here is small (N=3), these conclusions are supported by results from anesthetized macaques (N=5; Autio et al., 2021).

The similar inter-subject variability in FC between species was unexpected, particularly given that humans exhibit much greater variability in gyrification patterns than macaques (Hayashi et al., 2021). This finding suggests that neuroanatomical features like cortical folding may not be the primary drivers of variability in FC. Instead, it could suggest that neuronal activity patterns underlying FC are conserved across these species or are influenced by factors other than surface anatomy, such as macrovasculature (Autio et al., 2025; Kurzawski et al., 2022), CNR, synaptic architecture, or the intrinsic dynamics of neural circuits (Chaudhuri et al., 2015; Douglas & Martin, 2007). Indeed, neuronal high-frequency rhythms (Buzsáki et al., 2013) and neurovascular coupling mechanisms (Logothetis et al., 2001) are evolutionarily preserved and may translate into the similar temporal receptive fields and low-frequency vascular dynamics across primates.

The excellent reproducibility of the macaque data enables high-precision mapping of dense functional connectivity across sessions and subjects (Fig. 10B, 11B). In both species, we observed FC extending across sensorimotor and premotor areas (macaque: F1, 3a/b, 1, 2, 5, F2, and F5; human: M1, 3a/b, 1, 2, 7C, 6d, and 6v), the central proportion of the insula, and parietal cortex (macaque: proportions of areas SII, 7OP, and 7B; human PO). These results suggest that the dorsal proportion of macaque F5 may be a homologue of human area 6v, F2 may correspond to 6d and 5 to area 7C. In macaques, we identified two additional positively correlated nodes (9/46v and 9/46d) that were absent in the human resting-state FC maps (Fig. 11D). Anatomical and physiological evidence in macaques has linked areas 9/46v and 9/46d to complex motor planning, including arm movements (Barbas & Pandya, 1989; Hoshi & Tanji, 2004), and with similar functional evidence reported in humans (Beurze et al., 2007). It remains unclear whether this discrepancy stems from differences in data acquisition methods (CBVw vs. BOLD fMRI), differences in video presentation (macaques: dynamic Inscape; humans: static crosshair), analytic limitations (e.g., multivariate analysis, surface registration), or evolutionary differences. Despite these uncertainties, these homologous FC patterns provide a strong foundation for comparative neuroimaging and improved cortical landmarks for inter-species registration.

While our primary focus has been to demonstrate the ability to measure neural activity in the cerebral cortex, fMRI also offers a powerful tool for exploring subcortical circuits (Fig. 12B, C). Notably, subcortical structures exhibit superior FC in awake macaques than in anesthetized ones, likely due in part to the thalamus, which is particularly sensitive to anesthesia (Mapelli et al., 2021). This methodology, therefore, enhances our ability to investigate polysynaptic cortico-subcortical-cortical circuits, such as those involving the basal ganglia and cerebellar cortices—structures whose evolution remains poorly understood due to the lack of robust anatomical landmarks and the inherent challenges of studying polysynaptic circuits (Kelly & Strick, 2000; Magielse et al., 2023).

## 5 Conclusions

A 24-channel 3T receive coil was developed for accelerated neuroimaging of awake macaque monkeys. The use of accelerated ferumoxytol-weighted fMRI, combined with ICA-FIX artifact removal, produced highly reproducible parcellated functional connectivity patterns at the single-subject level. At the group level, dense seed-based functional connectivity exhibited neurobiologically robust and likely homologous networks in macaques and humans. The data acquisition hardware, imaging protocols, and data analysis pipelines presented here are publicly available, facilitating further exploration of the functional organization of primate brains by the broader scientific community.

## Supplementary Material

Supplementary Material

## Data Availability

The 24-channel RF receive coil for awake macaque monkeys is commercially available via Rogue Research, Montreal, Canada https://www.rogue-research.com/, produced by Takashima Seisakusho Co. Ltd. Tokyo, Japan. The imaging protocols are available at https://brainminds-beyond.riken.jp/hcp-nhp-protocol. The NHP version pipelines is now merged with HCP pipeline and available at https://github.com/Washington-University/HCPpipelines.

## Appendix

### Data Acquisition Strategy

To improve comparison of brain organization across primates, our data acquisition strategy is based on standardized minimal requirements, adjusting image resolution to the thinnest parts of the cerebral cortex (Autio et al., 2020; Glasser et al., 2013; Hayashi et al., 2021; Hori et al., 2018). The structural image resolution (0.5 mm) is approximately scaled to half of the minimum cortical thickness in macaques (≈1 mm) to ensure accurate reconstruction of the cortical pial and white matter surfaces. The fMRI spatial resolution (1.25 mm) is scaled below the 5^th^ percentile of cortical thickness (≈1.4 mm) to reduce partial volume effects from cerebrospinal fluid and white matter, and to improve differentiation between opposing banks of sulci (Autio et al., 2020; Glasser et al., 2013).

While our previous BOLD resting-state fMRI image acquisition protocol in anesthetized macaques closely followed HCP-YA’s acquisition protocols at 3T (Autio et al., 2020; Glasser et al., 2013), CBVw fMRI data acquisition in awake macaques requires different methodological considerations (Autio et al., 2021; Leite et al., 2002; Vanduffel et al., 2001). First, scan durations are more limited in awake macaques, necessitating the acquisition of data with high temporal sampling rates (e.g., short TR) for robust detection of functional networks. Second, in CBVw fMRI shorter TEs matched with contrast agent, dose-induced T_2_*=TE enhances contrast (see below) (Mandeville et al., 2004).

The intravascular injection of magnetic susceptibility contrast agent (e.g. ferumoxytol) effectively attenuates the intravascular blood water compartment and CSF nuisance signals located adjacent to major arteries and draining veins along the cortical surface (Autio et al., 2025; Zhao et al., 2006). Thus, the gray matter transverse relaxation rate ($R_{2}^{*}=1/T_{2}^{*}$) in CBVw fMRI can be expressed as a single-compartment model:

$$R_{2}^{*}=R_{2,int}^{*}+R_{2,shim}^{*}+R_{2,BOLD}^{*}+R_{2,ferumoxytol}^{*}\left(CBV,\;C\right),$$
(A1)

where $R_{2,int}^{*}$ corresponds to intrinsic relaxation processes, $R_{2,shim}^{*}$ represents susceptibility-induced B_0_ field inhomogeneities (e.g., near air–tissue interfaces), and $R_{2,BOLD}^{*}$ is (extravascular) blood oxygen level-dependent (BOLD) contrast, which is inversely proportional to CBV and the contrast agent dose, *C*. Here, $R_{2,ferumoxytol}^{*}$ denotes the contrast agent-induced increment in the transverse relaxation rate—that is, the increase in R2* relative to the pre-contrast (no-ferumoxytol) baseline—and is directly proportional to CBV and C (Mandeville et al., 2004).

In Eq. (2), signal loss due to the first three relaxation terms is of no interest and should be minimized, while the last contrast agent dose-dependent term should be maximized. Practically, this is achieved by minimizing TE and then optimizing the contrast agent dosage, thereby enhancing the relative contribution of $R_{2,ferumoxytol}^{*}$ (Mandeville et al., 2004). At spatial resolutions (1.25 mm isotropic), where the venous and arterial vessel contributions are not separable (Autio et al., 2025; Weber et al., 2008), it is crucial to use a contrast agent dose that overcomes the BOLD effect (so that $R_{2,ferumoxytol}^{*}$ >> $R_{2,BOLD}^{*}$), while maintaining sufficient tSNR to detect neural activity-associated signal fluctuations. The signal changes (ΔS) in resting-state fMRI can be expressed as a difference between the temporal average MRI signal and dynamic spontaneous fluctuations in CBV:

$$\Delta S=S_{average}-S_{dynamic},$$
(A2)

$$\begin{array}{c} \Delta S=S_{0}e^{-TE\left(R_{2,int}^{*}\;+\;R_{2,ferumoxytol}^{*}\right)}\\ \;\;\;-\;S_{0}e^{-TE\left(R_{2,int}^{*}\;+\;R_{2,ferumoxytol}^{*}\;+\;\Delta R_{2,ferumoxytol}^{*}\right)}, \end{array}$$
(A3)

where S_0_ represents the baseline MRI signal intensity at a TE of zero. Factoring out the average term yields

$$\Delta S=S_{0}e^{-TE\left(R_{2,int}^{*}\;+\;R_{2,ferumoxytol}^{*}\right)}\left(1-\;e^{-TE\left(\Delta R_{2,ferumoxytol}^{*}\right)}\right).$$
(A4)

Applying the first-order Taylor expansion (1 − e^−x^ ≈ x), we obtain

$$\Delta S\approx S_{0}e^{-TE\left(R_{2,int}^{*}\;+\;R_{2,ferumoxytol}^{*}\right)}\;\cdot \;TE\;\cdot \;\Delta R_{2,ferumoxytol}^{*}.$$
(A5)

Because the baseline and the dynamic change are linked by the intravascular ferumoxytol contrast agent concentration, scaling with underlying tissue microvascular properties ($R_{2,ferumoxytol}^{*}=C\;\cdot \;CBV$ and $\Delta R_{2,ferumoxytol}^{*}=C\;\cdot \;\Delta CBV$ , with the ferumoxytol relaxivity absorbed into *C*), we substitute these relationships to isolate and maximize the terms dependent on the contrast agent concentration, *C*:

$$\Delta S\approx S_{0}e^{-TE\;\cdot \;R_{2,int}^{*}}\cdot \;TE\;\cdot \;\Delta CBV\;\cdot \;C\;\cdot \;e^{-TE\;\cdot \;C\;\cdot \;CBV}$$
(A6)

$$f\left(C\right)=C\;\cdot \;e^{-TE\;\cdot \;C\;\cdot \;CBV}.$$
(A7)

Taking the derivative with respect to C and setting it to 0:

dfdC= e−TE · C · CBV+C · (−TE · CBV · e−TE · C · CBV)

$$=\;e^{-TE\;\cdot \;C\;\cdot \;CBV}\left(1-TE\;\cdot \;C\;\cdot \;CBV\right)=0.$$
(A8)

Since the exponential term *e^−TE ∙ C ∙ CBV^* ≠ 0, the maximum occurs when:

1−TE · C · CBV=0,
(A9)

which gives

$$C\;\cdot \;CBV=1\frac{}{}TE.$$
(A10)

Substituting back the baseline relation ($R_{2,ferumoxytol}^{*}$ = *C · CBV*), the optimum contrast condition is established when relaxation rate satisfies

$$R_{2,ferumoxytol}^{*}=1\;/\;TE.$$
(A11)

It is worth noting that if the competing BOLD effect ($-R_{2,BOLD}^{*}$) accompanying spontaneous ∆CBV increases is explicitly accounted for, the mathematical optimum shifts slightly higher to $1/TE\;+\;R_{2,BOLD}^{*}$. However, at the high ferumoxytol doses utilized here, the contrast agent-driven susceptibility effects heavily overwhelm the intrinsic BOLD changes, effectively keeping the peak sensitivity target at the classic 1/TE boundary (Mandeville et al., 2004). Finally, extending this framework to optimize the CNR (= ΔS / σ_N_) yields an identical result. Assuming independent, additive background noise (σ_N_) that is unaffected by the contrast agent dose, maximizing CNR is mathematically equivalent to maximizing raw signal variance.
