# Supplementary material for: Development of a 24-channel 3T phased-array coil for fMRI in awake monkeys: Mitigating spatiotemporal artifacts in ferumoxytol-weighted functional connectivity estimation
Source: Imaging Neurosci (Camb). 2026 Sep 3;4:IMAG.a.1354. doi: 10.1162/IMAG.a.1354 (PMC13543443; doi:10.1162/IMAG.a.1354)
Supplement: Supplementary file 1 [file IMAG.a.1354_supp.pdf]

Supplementary Materials

Development of a 24-Channel 3T Phased-Array Coil for fMRI in Awake Monkeys: Mitigating Spatiotemporal Artifacts in Ferumoxytol-Weighted Functional Connectivity Estimation

Authors

Joonas A. Autio[1,6], Atsushi Yoshida[1,2], Yoshihiko Kawabata[3], Masahiro Ohno[1], Kantaro Nishigori[1,4], Takayuki Ose[1], Stephen M. Smith[5], David C. Van Essen[6], Matthew F. Glasser[6,7], Takuya Hayashi[1,8,9]

Affiliations

[1]Laboratory for Brain Connectomics Imaging, RIKEN Center for Biosystems Dynamics Research, Kobe, Japan

[2]Systems Neuroscience Laboratory, Hokkaido University Graduate School of Medicine, Sapporo 060-8638, Hokkaido, Japan

[3]Takashima Seisakusho Co., Ltd, Tokyo, Japan

[4]Sumitomo Pharma Co., Ltd, Osaka, Japan

[5]Centre for Integrative Neuroimaging, FMRIB, Nuffield Department of Clinical Neurosciences,University of Oxford, UK

[6]Department of Neuroscience, Washington University Medical School, St Louis, MO USA

[7]Department of Radiology, Washington University Medical School, St Louis, MO USA

[8]Department of Brain Connectomics, Kyoto University Graduate School of Medicine, Kyoto, Japan

[9]Section of Brain Function Information, National Institute for Physiological Sciences. Okazaki, Japan

Corresponding author

Takuya Hayashi, MD, PhD

Laboratory for Brain Connectomics Imaging

RIKEN Center for Biosystems Dynamics Research

6-7-3 MI R&D Center 3F, Minatojima-minamimachi

Chuo-ku, Kobe 650-0047, Japan

E-mail: takuya.hayashi@riken.jp

Including five figures.

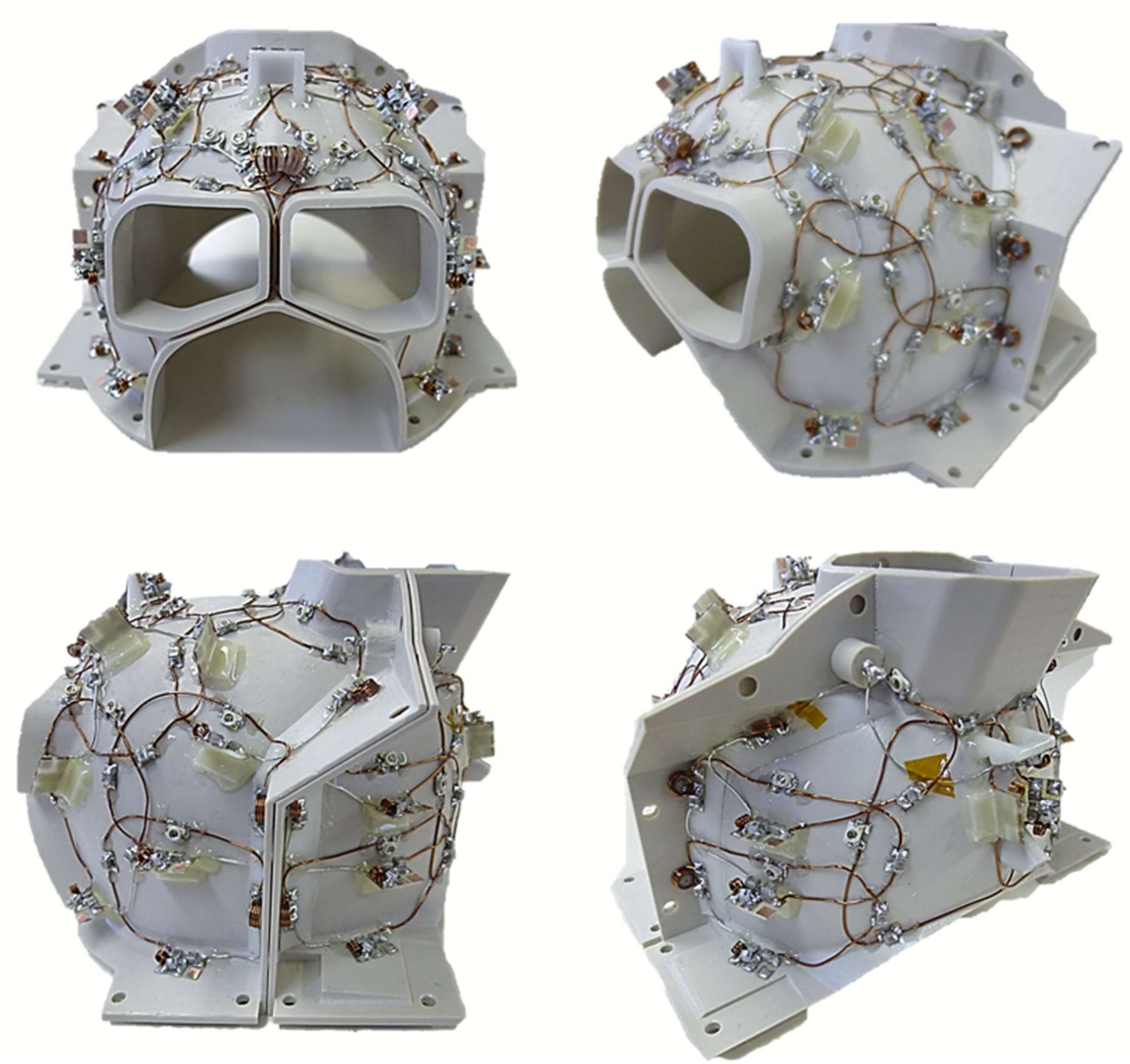

Supplementary Figure 1. Additional views of the 24-channel phased-array macaque coil.

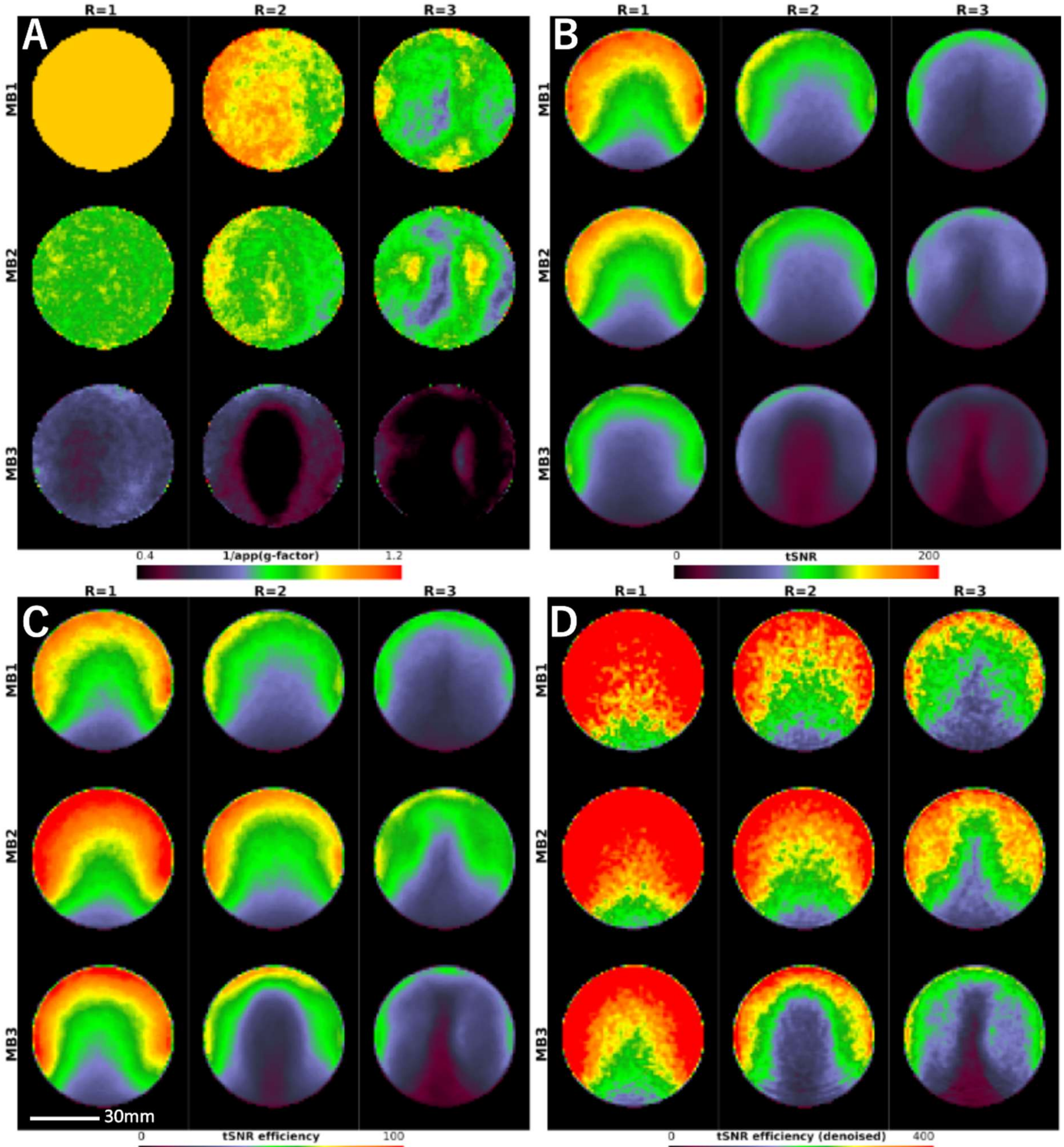

Supplementary Figure 2. Evaluation of in-plane Generalized Autocalibrating Partially Parallel Acquisitions (GRAPPA) and out-of-plane multiband (MB) acceleration effects on temporal signal characteristics using the anesthetized macaque coil (Autio et al., 2020). (A) Inverse geometry-factor (1/g) maps as a function of GRAPPA reduction (R) and MB acceleration factors. **(B)** Temporal signal-to-noise ratio (tSNR) across varying combinations of R and MB. **(C)** tSNR efficiency per unit time, computed as tSNR normalized by the square root of the repetition time. **(D)** Post-ICA tSNR efficiency maps demonstrating that combined MB and GRAPPA acceleration preserves signal quality in peripheral regions (approximately corresponding to the cerebral cortex), with moderate signal attenuation in central brain regions. Note that the phantom was placed in exactly the same orientation with respect to the magnet bore as in the assessment of the current awake macaque coil (Fig. 4) in order to achieve the same B_0 biasfiled and image distortion between the two conditions. In contrast, the anesthetized macaque coil was placed as if for an (anesthetized) macaque in the head-first-supine position, whereas the awake macaque coil (see Fig. 4) as if for an alert macaque in the head-first-sphinx position. Thus, different orientation of the phantom with respect to the coil results in the different bias in the distribution of signal sensitivity. The signal attenuation observed at the bottom of the images in this figure is due to the bottom part of the phantom being furthest away from the receive coil elements, whereas this part of the phantom was very close to them and showing higher signals in the awake macaque coil (Fig. 4).

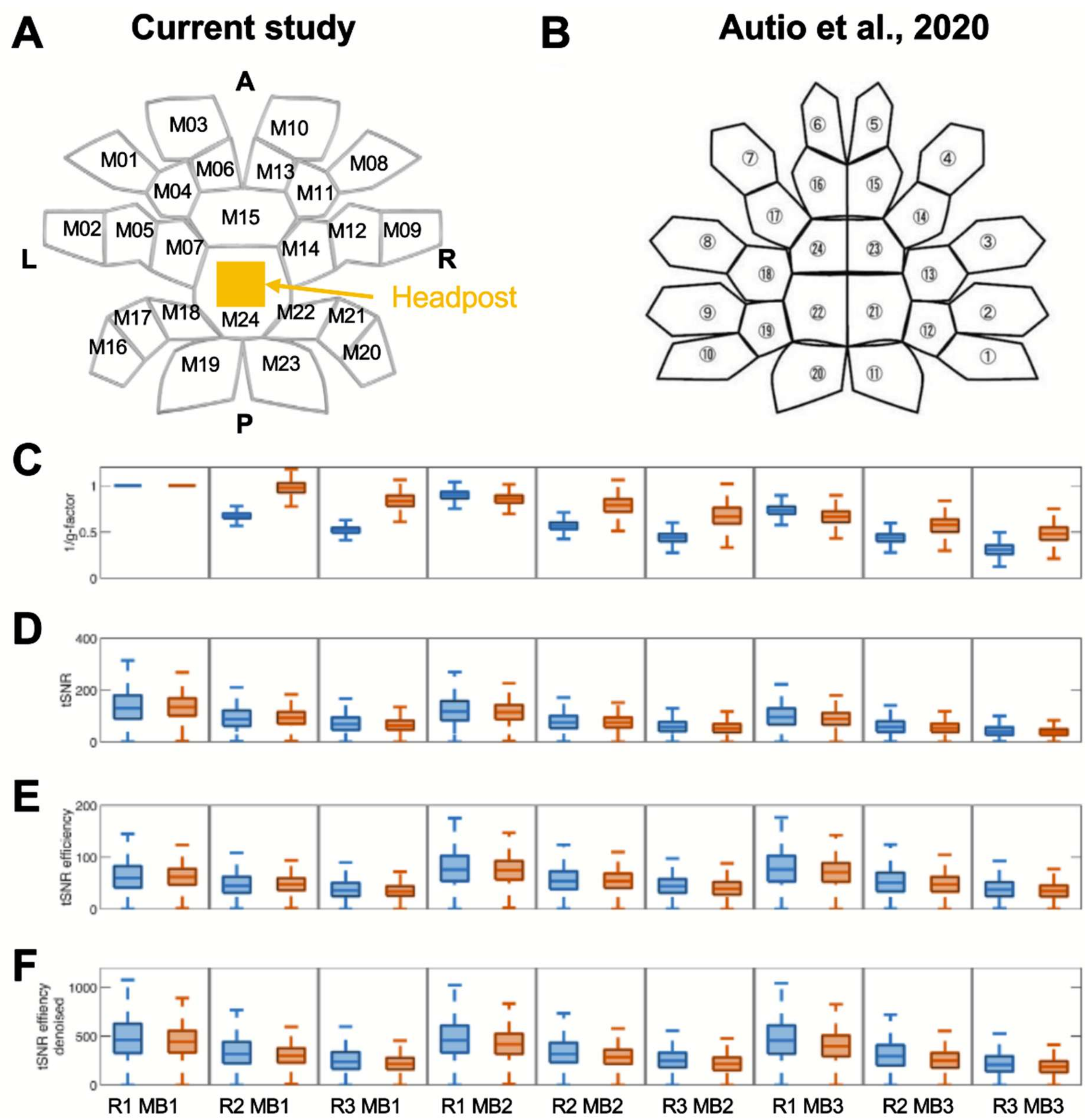

Supplementary Figure 3. Comparison between the newly designed head-post compatible modular awake coil (current study) and previous coil design for anesthetized macaque monkeys (Autio et al., 2020). (A, B) Coil element wiring maps. The awake coil design includes a centrally positioned, large-diameter element (M24) to accommodate rigid headpost attachment. In addition, the awake coil can be easily separated into anterior and posterior halves using an inductor-based overlapping method. Box-plots of **(C)** Inverse geometry-factor (1/g), **(D)** Temporal signal-to-noise ratio (tSNR), **(E)** tSNR efficiency per unit time, and **(F)** ICA denoised tSNR efficiency maps. The blue and orange boxplots correspond to the awake coil and anesthetized coils, respectively. Abbreviations: R: GRAPPA Reduction factor; MB: Multiband factor.

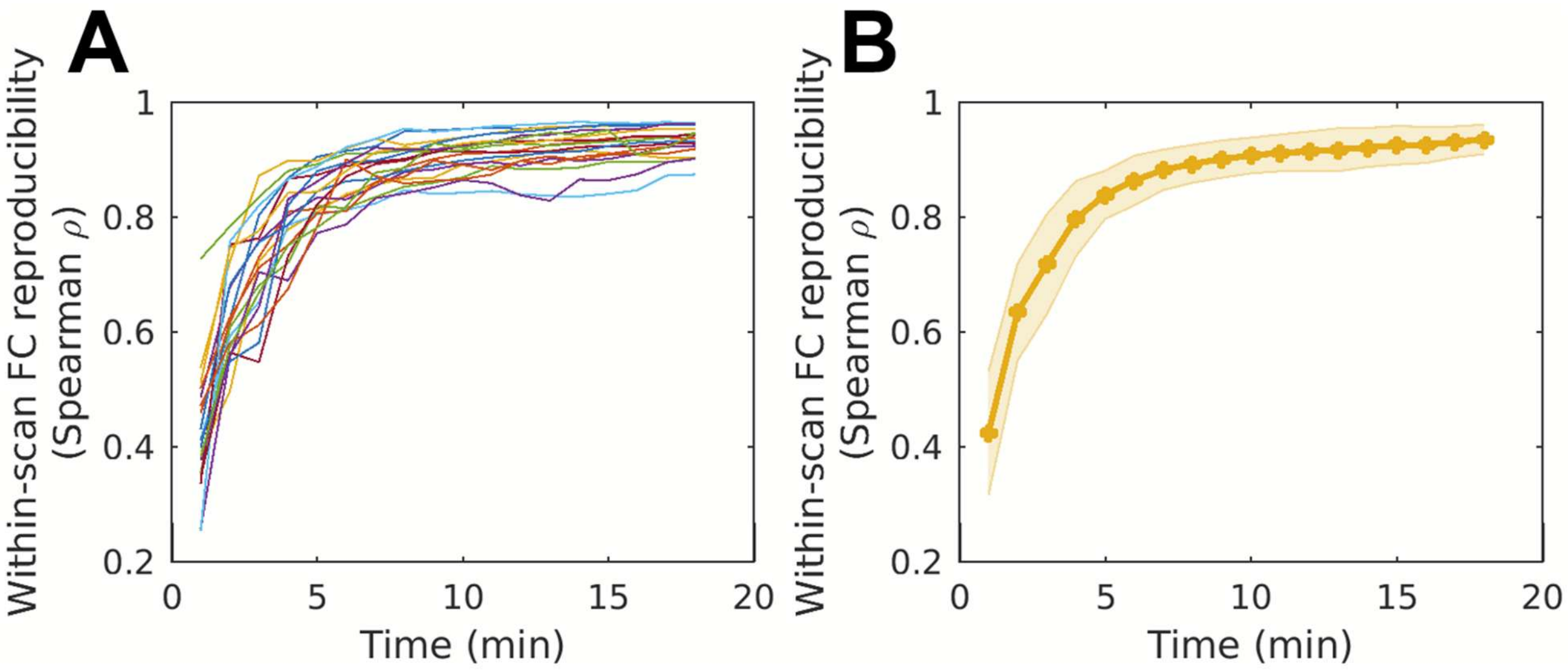

Supplementary Figure 4. Within-scan reproducibility of M132 parcellated functional connectivity matrix. **(A)** Reproducibility of within-scan M132 atlas-parcellated functional connectivity correlation matrix plotted as a function of scan duration. Each color indicates a separate scan session. **(B)** On average, within scan reproducibility reached excellent quality (Spearman Rho > 0.90) using a pair of 10 minute scans.

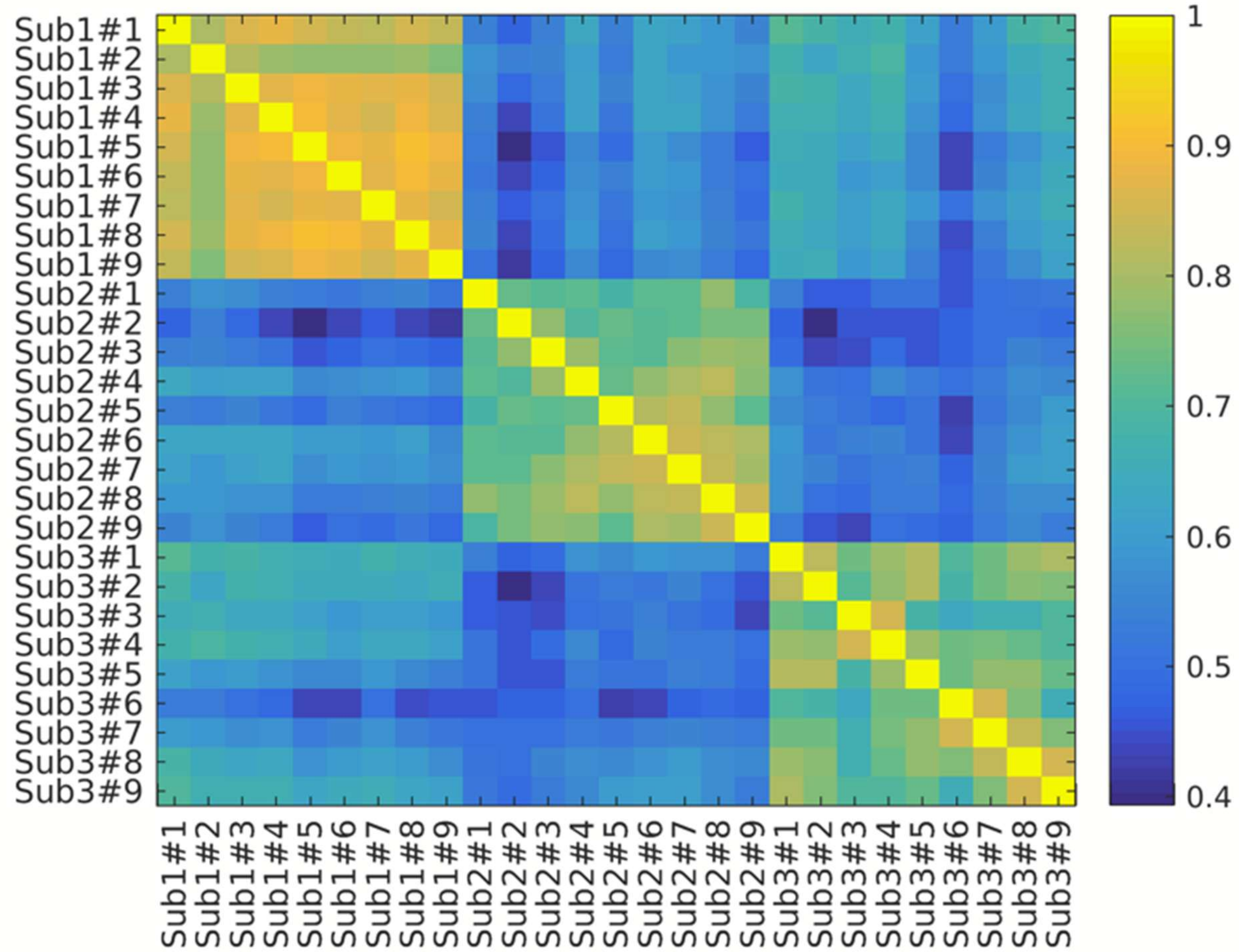

Supplementary Figure 5. Identifying subjects using noise-dominant correlation patterns.
